# Lipid Profile of *Xylella fastidiosa* Subsp. *pauca* Associated With the Olive Quick Decline Syndrome

**DOI:** 10.3389/fmicb.2018.01839

**Published:** 2018-08-14

**Authors:** Valeria Scala, Massimo Reverberi, Manuel Salustri, Nicoletta Pucci, Vanessa Modesti, Simone Lucchesi, Stefania Loreti

**Affiliations:** ^1^Centro di Ricerca Difesa e Certificazione, Consiglio per la Ricerca in Agricoltura e l’Analisi dell’Economia Agraria, Rome, Italy; ^2^Dipartimento di Biologia Ambientale, Sapienza University of Rome, Rome, Italy

**Keywords:** lipidome, bacterial plant pathogen, diffusible factors, oxylipins, free fatty acid, lipid signals

## Abstract

Lipids, components of the plasma and intracellular membranes as well as of droplets, provide different biological functions related to energy, carbon storage, and stress responses. Bacterial species display diverse membrane composition that changes in response to the different environmental conditions. During plant–pathogen interactions, lipids might have roles in several aspects such as recognition, signal transduction, and downstream responses. Among lipid entities, free fatty acids (FFAs) and their oxidized form, the oxylipins, represent an important class of signaling molecules in host–pathogen perception, especially related to virulence and defense. In bacteria, FFAs (e.g., diffusible signaling factors) and oxylipins have a crucial role in modulating motility, biofilm formation, and virulence. In this study, we explore by LC-TOF and LC-MS/MS the lipid composition of *Xylella fastidiosa* subsp. *pauca* strain De Donno in pure culture; some specific lipids (e.g., ornithine lipids and the oxylipin 7,10-diHOME), characteristic of other pathogenic bacteria, were revealed. *Nicotiana tabacum* was used for testing the ability of this pathogen in producing such lipids in the host. Different lipid compounds present a clear distribution pattern within the infected plant tissues compared to the uninfected ones.

## Introduction

Among bacterial plant pathogens, *Xylella fastidiosa* is one of the most dangerous bacteria causing devastating diseases and showing an extensive natural host range. *X. fastidiosa*, with diverse modalities related to the subspecies and the host, causes different diseases such as Pierce’s Disease (PD) of grapevine, phony peach, leaf scald of plum, citrus variegated chlorosis, olive quick decline, and leaf scorch in almonds, coffee, and oleander (Wells et al., 1987; Hopkins, 1989). Recently, Saponari et al. (2017) assessed the pathogenic role of *X. fastidiosa* subsp. *pauca* strain De Donno in olive and other susceptible host plants.

Lipids play important roles at various stages of host–pathogen interactions (van der Meer-Janssen et al., 2010; Siebers et al., 2016; Sohlenkamp and Geiger, 2016) and are crucial in determining the virulence of bacterial pathogens (Martinez and Campos-Gomez, 2016). Free fatty acids (FFAs) might also function as modulators of several pathways in bacterial cell-to-cell communication such as the diffusible signaling factor (DSF). Notably, DSF acts as regulator of biofilm formation and as virulence factor in several plant bacterial pathogens, as for instance *X. fastidiosa* (Dow, 2017). In *X. fastidiosa*, the DSF family can also participate in the inter-kingdom communication with plants or insects (vector) (Ionescu et al., 2016). Unsaturated fatty acids (FAs) may also act as substrates for oxidizing enzymes [e.g., lipoxygenases (LOXs) and dioxygenases (DOXs)] forming oxylipins that have been extensively studied in plant–pathogen interaction (Blèe, 2002; Christensen and Kolomiets, 2011). The oxylipins, *per se* or conjugated with sugars and aminoacids, are bioactive molecules; the oxylipin jasmonic acid and its derivatives in plants mediate hormone-like functions and are involved in defense responses (Jones and Dangl, 2006). Notwithstanding their importance, the role of oxylipins is almost underestimated and understudied in phytopathogenic prokaryotes. Very recently, Martinez and Campos-Gomez (2016) show that the opportunistic bacterial pathogen *Pseudomonas* *aeruginosa* may transform monounsaturated FAs into mono- and di-hydroxylated derivatives during its interaction with the host (e.g., lettuce). In this pathogen, the oleic acid-derived oxylipins negatively control the motility of flagella, cause the upregulation of twitching motility and promote bacterial organization in micro colonies and the formation of biofilms *in vitro* and *in vivo*, controlling the virulence in the host (Martinez and Campos-Gomez, 2016).

Lipids play important roles in plant disease (Siebers et al., 2016; Sohlenkamp and Geiger, 2016); the membrane lipid compositions can vary among bacterial species depending on the environmental conditions to which the organism is exposed (Giles et al., 2011; Lewenza et al., 2011; Vences-Guzmán et al., 2011; Moser et al., 2014; Sohlenkamp and Geiger, 2016). Glycerophospholipids of the bacterial membranes present a hydrophobic tail composed by two FAs, a glycerol backbone, a hydrophilic head of phosphate and groups such as phosphatidylethanolamine (PE), phosphatidylglycerol (PG), cardiolipin (CL), lysyl-phosphatidylglycerol (LPG), phosphatidylinositol (PI), phosphatidic acid (PA), and phosphatidylserine (PS). Bacterial membranes present also phosphorus-free lipids and notably ornithine/glutamine lipids (O/GlnLs), diacylglyceryl-N,N,N-trimethylhomoserine (DGTS), sulfolipids, monoacylglycerol (MAG), diacylglycerol (DAG), triacylglycerol (TAG), glycolipids (GLs), and hopanoids (BHPs) (Sohlenkamp and Geiger, 2016). Phospholipids can be replaced within the membrane lipids of bacteria with phosphorus-free lipids; this remodeling has been thoroughly studied in *Rhodobacter sphaeroides*, *Sinorhizobium meliloti*, *Agrobacterium tumefaciens*, and *Mesorhizobium loti* (Benning et al., 1995; Geiger et al., 1999; Zavaleta-Pastor et al., 2010; Devers et al., 2011; Geske et al., 2013; Diercks et al., 2015). Bacterial phosphate-free membrane lipids and in particular OLs and their hydroxylated forms, are important for interaction with plants (Vences-Guzman et al., 2013). Some bacteria form OLs only under phosphorous-limiting conditions; in others, OLs are formed constitutively. Vences-Guzman et al. (2015) estimate that about 50% of the bacteria can produce OL. A mutant of the plant pathogen *Agrobacterium fabrum* (formerly: *A. tumefaciens* C58), lacking hydroxy-OL or any OLs, anticipates the formation of tumors that are even bigger than those produced by the wild type upon plant infection. Vences-Guzman et al. (2013) hypothesize that the recognition of OL or hydroxy-OL might elicit plant defense responses; *A. fabrum* devoid of OL or hydroxy-OL, escape the plant immune system thus inducing an accelerated infection process. In *Rhizobium tropici* and *Burkholderia cepacia*, the 2-hydroxylation of OL is also involved in the growth at higher temperature condition (Taylor et al., 1998; Vinuesa et al., 2003; Vences-Guzmán et al., 2011). Among the phosphorus-free membrane lipids, BHPs are present in a wide variety of *prokaryota* and have structural similarities with eukaryotic sterols (Saenz et al., 2012). BHPs enhance the stability and impermeability of the bacterial membranes. Strains of *Burkholderia cenocepacia*, defective in BHPs production, display increased sensitivity to low pH, detergents, and various antibiotics and cannot produce flagella (Schmerk et al., 2011). Within phosphorous-containing lipids, some authors demonstrated that PC, which in bacterial membranes might account up to > 20% of total phospholipids (Klüsener et al., 2009), are involved in the virulence of *Agrobacterium tumefaciens*. Deletion of *A.* *tumefaciens* *pmtA* partly impaired the synthesis of PC, delayed tumor formation that is reduced in size (Wessel et al., 2006). Furthermore, the authors highlight that tumor formation is absent when the host is infected with the PC-free double mutant Δ*pmtA* and Δ*pcs* of *A. tumefaciens*. *Pseudomonas syringae* pv. *syringae* requires PC for virulence and specifically for secreting, through the type III secretion system, the effector HrpZ (Xiong et al., 2014).

In phytopathogenic bacteria, different type of lipids may drive the compatibility or incompatibility with the host. In relation to this, the present study first investigates by mass spectrometry the lipid composition of *X. fastidiosa* subsp. *pauca* strain De Donno under *in vitro* conditions. This approach allows individuating and identifying several lipid compounds produced by this pathogen in the cell as well as in the culture media. Second, the model plant *Nicotiana tabacum* was inoculated with this bacterial pathogen. Mass spectrometry analysis shows a differential accumulation of lipid entities among infected and non-infected plants; notably, some FFAs, complex lipids and oxylipins could play a role during plant colonization.

## Materials and Methods

### Chemicals

The HPLC/MS-grade methanol (MeOH) and isopropyl alcohol (iPrOH) were purchased from Merck (Darmstadt, Germany), and HPLC/MS grade ammonium formate (HCOONH_4_) was purchased in granular form from Fluka (Buchs SG, Switzerland). Authentic tricosanoic acid (C23:0; MW 355.25 g/mol), deuterated 9-hydroxy-10E,12Z-octadecadienoic acid (9-HODEd_4_, MW 300.5 g/mol) used as the internal standards (IS), behenic acid (C22:0; MW 340.33), erucic acid (C22:1; MW 338.31), arachidic acid (C20:0; MW 304.24), eicosenoic acid (C20:1; MW 310,28), eicosadienoic acid (C20:2; MW 308.27), non-adecanoic acid (C19:0; MW 298.28), stearic acid (C18:0; MW 284.43), oleic acid (C18:1; MW 282.5), linoleic acid (C18:2; MW 280.45), linolenic acid (C18:3; MW 278.43), heptadecanoic acid (C17:0; MW 270.25), palmitic acid (C16:0; MW 256.42), palmitoleic acid (C16:1; MW 254.41), pentadecanoic acid (C15:0; MW 242.22), tetradecanoic acid (C14:0; MW 228.20), tridecanoic acid (C13:0; MW 214.34), dodecanoic acid (C12:0; MW 200.32), undecanoic acid (C11:0; MW 186.29), trioleoylglycerol (TAG 54:3; MW 884.78), 1,2-dioleoylglycerol (DAG 36:2; MW 620.99), palmitoyl-2-glycerol (MAG 16:0; MW 330.5), 1,2-Dipalmitoyl-*sn*-glycero-3-phospho-*rac*-(1-glycerol) (PG 32:0; MW 722.5), 1,2-dinonadecanoyl-*sn*-glycero-3-phosphocholine (PC 38:0; MW 818.2), and 1,2-diheptadecanoyl-*sn*-glycero-3-phosphoethanolamine (PE 34:0; MW 719.5) were purchased from Sigma-Aldrich (St. Louis, United States) and Avanti Polar Lipids (Alabaster, United States). Bacteriohopane-32,33,34,35-tetrol (BHT; MW 547.47) and ornithine lipids OL3-OH (18:1/19:1) (MW 691.2) were kindly provided by Dr. Rachel Schwartz-Narbonne (Newcastle University, United Kingdom) and by Dr. Christian Sohlenkamp (Universidad Nacional Autónoma de México). The mixture of standards was dissolved in acetone/MeOH/iPrOH 40/40/20 at the final concentration 2 μM.

### Bacterial Strains and Culture Conditions

*Xylella fastidiosa* subsp. *pauca* strain De Donno (CFBP 8402) (from here XfCFBP8402) grown for 7 days at 28°C in buffered charcoal yeast extract (BCYE) was harvested from the medium (Wells et al., 1987) and suspended in sterile potassium phosphate buffer (0.05 mM pH 7.2; PBS) to a final concentration of approximately OD_600_ of 0.5, corresponding to ca. 10^8^ colony forming unit CFU/mL, as reported in Giampetruzzi et al. (2016). Aliquots (100 μL) of this bacterial suspension were used to inoculate PD2 liquid medium (Davis et al., 1980) and grown for 7 and 11 days at 28°C and 100 rpm. A total of 10 independent experiments (*n* = 6 in each experiment) for the lipid extraction were performed.

### *Nicotiana tabacum* Inoculation

*Nicotiana tabacum* “Petite Havana SR1” were propagated in a greenhouse as reported in the European Plant Protection Organization (EPPO) Standard PM 7/24 (2) (European Plant Protection Organization, 2016) and prepared before inoculation as described previously (Francis et al., 2008). Briefly, the apical part of the stem was cut while removing the lower juvenile leaves, thus remaining the sole three adult leaves (numbered as 1–3) in the lower portion of the plant. The plants were inoculated with XfCFBP8402 and control plants (named mock) were treated only with the buffer. The inoculum concentration was prepared at approximately 10^9^ CFU/mL. The infections were performed at least in three independent experiments, each comprising infected (*n* = 20) and mock plants (*n* = 20). Each plant was inoculated (20 μL bacterial suspension or buffer) in the petioles near the axils of the three healthy adult leaves 1–3. A total of 15 days after inoculation (DAI), the petiole of the first leaf (numbered as 4) above leaves 1–3 was collected from each plant and the DNA was extracted according to the modified DNeasy^®^ Mericon TM Food standard kit (Qiagen) following manufacturers’ instructions. The presence of bacterial DNA was confirmed by real time PCR as indicated by Harper et al. (2010). Among the infected specimens, only the plants positive to XfCFBP8402 were selected for further analysis. The same real time PCR procedure was performed to confirm the mocks as XfCFBP8402-negative. A total of 30 DAI, the first two leaves (numbered as 5 and 6) upon the leaf 4 were collected from at least 15 XfCFBP8402-positive plants (from here named as “Xf-infected”) and grouped in a bulk. The samples were separated in two parts: one (from here named “petiole”) composed by the petioles and central vein and the other (from here named “leaf”) composed of the marginal leaf. Petioles and leaves were lyophilized and ground with liquid nitrogen. The same collection procedure was followed for the mock plants. The bacterial DNA amount in the petioles and leaves of XfCFBP8402-infected samples as well as in the mock samples was evaluated as reported in Modesti et al. (2017). In particular, DNA was extracted using the modified DNeasy^®^ Mericon TM Food standard kit (Qiagen) following the manufacturers’ instructions and amplified by quantitative real time PCR (Harper et al., 2010). Lipid extraction from the petioles and leaves of XfCFBP8402-infected and mock plants was performed as described below.

### Lipid Extraction

Lipids were extracted from pelleted cells and lyophilized culture filtrate of XfCFBP8402 at different DAI (7–11 DAI), and from tobacco plants, Xf-infected and mock, at 30 DAI. The internal reference standards tricosanoic acid and 9-HODEd_4_, for the analysis were added at a final 2-μM concentration. The extraction was performed on pelleted bacterial cells or 30 mL of lyophilized culture filtrate or 20 mg of lyophilized plant material that were extracted with 2 mL of iPrOH: H_2_O: EtOAc (1:1:3 v/v); butylated hydroxytoluene (0.0025% w/v) was added to avoid oxidation. After centrifugation, the ethyl acetate upper phase was collected in a clear tube and dried with nitrogen. The extraction was repeated on the initial matrix adding 1.2 mL of EtOAc and then vortex-mixing. After centrifugation, the upper phase was recovered and transferred to the collection tube together with the previously extracted fraction, and dried under nitrogen flux. The dried samples were resuspended in MeOH (100 μL).

### Nomenclature and Abbreviations Used to Describe Lipid Components

Notation of lipids common to eukaryote and prokaryote such as MAG, DAG, TAG, glycerophosholipids was done as reported in Camera et al. (2010). In relation to bacterial-specific lipids, BHPs was used for indicating the hopanoids while OL was the abbreviation for ornithine lipids (OLs). In these bacterial lipids, the ornithine head group is bound *via* its N_α_–amino group to a 3–OH FA (R_1_), with a second FA chain (R_2_) esterified to the 3–OH group of the first FA. Thus, OL3-OH (18:1/19:1) indicates an ornithine esterified with an oleic acid that is subsequently esterified with a non-adecenoic acid (Sohlenkamp and Geiger, 2016). Notation for FAs and oxylipins (OM/D/TrE) is reported as indicating the carbon number (CN) and the number of double bond (DB) equivalents (e.g., C18:1 were oleic acid and HODE hydroxyoctadecenoic, respectively). The short notation for free FA is FFA (Liebisch et al., 2013).

### Lipid Analysis

The HPLC runs and accurate mass measurements of lipids were conducted with a G6220A TOF-MS, (Agilent Technologies, United States) equipped with an electrospray interface operating in the negative as well as in positive ion scan mode (m/z 100 1200) as indicated in Camera et al. (2010) (**Supplementary Figures S1A,B**). Our attention was focused on specific classes of lipids and namely: MAG, DAG, TAG, BHP, OL, some of the most represented phospholipids (i.e., PC, PE, and PG), FFA, and oxylipins.

Some lipid classes were further analyzed (fragmentation analysis) by liquid chromatography (HPLC 1200 series rapid resolution) coupled to the triple quadrupole G6420A (Agilent Technologies, United States) equipped with an electrospray ionization (ESI) source. The same elution conditions (phases, gradient, and column) described above were used.

Several lipid classes were searched through neutral loss (NL) and/or precursor ion mode in the XfCFBP8402 pelleted cells, culture filtrate and plant samples. Specifically, PC can be identified by scanning in positive ion mode for precursors of m/z 184.2 while dehydrated OLs at m/z 115.2 (**Supplementary Table S1A**). By scanning precursors in negative ion mode it was possible to individuate several species of OLs (precIon at m/z 131.2, Zhang et al., 2009; Moore et al., 2016). PEs were searched by NL at m/z 141.1 (Brügger et al., 1997). Product ion (PI) scan mode was used for characterizing mass fragmentation pattern of several lipid compounds (**Supplementary Table S1B**). The energies of collision were tailored upon each PI of interest. PE, PC, MAG, OL, and BHP were analyzed in positive ionization with [M+H]^+^ parent ion. DAG and TAG were analyzed in positive ionization with [M+NH_4_]^+^ parent ion. PG were analyzed in negative ionization with [M−H]^−^ parent ion.

The single ion monitoring (SIM) analysis aided us in individuating and quantifying the FFA (**Supplementary Table S1C**) in the samples. Standard curves in the range of 0.1 μM to 4 mM were originated for each FA listed in **Supplementary Table S1C**. Rate was linear within the range and regression curves [limit of detection (LOD) and limit of quantification (LOQ)] were originated to calculate the exact amount of each FA in real samples (**Supplementary Table S2**). Further, in plant samples, a multiple reaction monitoring (MRM) method (with the same chromatographic settings) was adopted to analyze the most abundant lipid entities in bacterial cells; the fragmentor voltage (F) and collision energies (CE) were optimized for each compound as previously described. Bacterial and plant *s*amples were analyzed for the presence of oxylipins as reported in Ludovici et al. (2014). Flow injection of authentic standards comparison with the literature (Yang et al., 2009; Strassburg et al., 2012; Ludovici et al., 2014; Martinez and Campos-Gomez, 2016) was used to confirm MRM transitions; for some compounds not reported in previous studies, fragmentation patterns were identified in bacterial cell extracts (e.g., jasmonates; Balcke et al., 2012; **Supplementary Figure S2**).

The MRM data were processed using the Mass Hunter Quantitative software (B.07.00 version) (**Supplementary Table S3A**). In **Supplementary Table S3B**, transitions, collision energy (CE), and fragmentor voltage (F) values are reported for each oxylipin analyzed. The putative structures of the main oxylipins found in our samples are shown in **Supplementary Table S3C**.

### Statistics

Average RT, peak areas, and mass accuracy of identified lipids were calculated for the analyzed samples as indicated in Camera et al. (2010). XLSTAT Version 2015.3.01.19199 (Addinsoft, Paris, France) as statistic package. In each experiment, datasets were pooled and compared using Student’s *t*-test, and the differences were considered significant when the *p*-value was < 0.05.

## Results

### Lipid Profile

The lipidomic profile of cells and cultural filtrate of XfCFBP8402 was acquired at different time intervals of bacterial growth, i.e., 7 and 11 DAI in liquid media (see the Section “Materials and Methods”). Results of TIC chromatogram are reported in **Supplementary Figures S1A,B**. LC-TOF chromatograms were searched for specific classes of lipids namely, MAG, DAG, TAG, OL, PE, PC, BHPs, FFA, and oxylipins. The analysis was performed basing on referenced data (e.g., Talbot et al., 2003; Han, 2016; Sohlenkamp and Geiger, 2016, *inter alia*), databases^1^^,^^2^ and by comparison with authentic standards. To assign properly the entities found we followed an approach exploiting MS/MS analysis.

### Identification of Acylglycerols

#### Triacylglycerides

Authentic trioleoylglycerol (TAG 54:3) [M+NH_4_]^+^ ions were generated under our chromatography conditions (see the Section “Materials and Methods”); its elemental composition was C_57_H_104_NO_6_ (*m/z* 902.8175). The extracted ion chromatogram (EIC) of *m/z* 902.8175 (**Supplementary Figure S3A**) for TAG 54:3 showed isobaric forms in XfCFBP8402 (cell extracts at 7–11 DAI) at comparable RT (21.6 min). Accordingly to Fauconnot et al. (2004), MS/MS fragments in the PI scans of the standard TAG 54:3 were due to the NL of RCOONH_4_ for each acyl chain (*m/z* 603.4) and minor fragments attributable to the oleic acid (**Supplementary Figure S3B**). PI scans of ions with *m/z* 902.8 (**Supplementary Figure S3C**) in XfCFBP8402 cell extracts confirmed their identity as TAG 54:3. TOF analysis showed that the TAG present into XfCFBP8402 cells eluted in the time range 20.9 ± 0.02 to 22.1 ± 0.06 min and ranged from *m/z* 694.5981 ± 0.0011 (C_42_H_80_NO_6_, TAG 39:2) to *m/z* 904.8328 ± 0.0005 (C_57_H_110_NO_6_, TAG 54:2). In relation to this, we identified at least 18 TAG families having 0 to 5 DBs (**Table 1**). Most of the peaks of TAG presenting an asymmetrical peak shape suggested the presence of isobaric TAG species (data not shown) that could derive either from different acyl chains [same acyl chains number (ACN) and DB] or from TAG regioisomers (Fauconnot et al., 2004). In addition to TAG 54:3, other two TAGs (52:2; 48:0) were fragmented for confirming their identities (**Supplementary Figures S3D,E**). Based on their fragmentation, these TAGs were tentatively assigned as 16:0/18:1/18:1 and 15:0/16:0/17:0, respectively.

**Table 1:** Most abundant TAG molecular ions detected in *X. fastidiosa* subsp. *pauca* strain De Donno (abundance cutoff at 10^3^ counts relative intensity).

	DB:0		DB:1		DB:2		DB:3		DB:4		DB:5	
	[M+NH_4_]^+^	RT	[M+NH_4_]^+^	RT	[M+NH_4_]^+^	RT	[M+NH_4_]^+^	RT	[M+NH_4_]^+^	RT	[M+NH_4_]^+^	RT
TAG 39					694.5981	20.9						
TAG 40					708.6137	21.1						
TAG 46			794.7232	21.2								
TAG 48	**824.7702**	**21.7**	822.7545	21.5	820.7389	21.2	818.7232	21.0				
TAG 50			850.7858	21.8	848.7702	21.5	846.7545	21.3				
TAG 52			878.8171	22.1	**876.8015**	**21.8**	874.7858	21.6	872.7702	21.4		
TAG 54					904.8328	22.1	**902.8175**	**21.9**	900.8025	21.7	898.7858	21.5

*MS/MS experiments have been performed on the m/z highlighted in bold font.*

#### Diacylglycerides

The elemental composition of authentic 1,2-dioleoylglycerol (DAG 36:2) was C_39_H_76_NO_5_ (*m/z* 638.5723). In XfCFBP8402 cell extracts (7–11 DAI), DAGs isobaric with the standard were present at comparable RT (18.8 min; **Supplementary Figure S4A**). In agreement with previous reports (Murphy et al., 2007; Hutchins et al., 2008; Camera et al., 2010), DAG 36:2 provide specific fragments caused by the NL of RCOONH_4_ for each acyl chain (oleic acid; *m/z* 282.4614) (**Supplementary Figure S4B**). PI scans of ion with *m/z* 638.5723 in the pooled samples of XfCFBP8402 cell extracts (7–11 DAI) confirmed its identity as DAG species (18:1/18:1) (**Supplementary Figure S4C**); in these samples, DAG were eluted between 9.4 ± 0.05 and 19.4 ± 0.07 min with [M+NH_4_]^+^ ions ranging from *m/z* 554.4784 ± 0.0008 (C_33_H_64_NO_5_, DAG 30:2) to *m/z* 642.6036 ± 0.0006 (C_39_H_80_NO_5_, DAG 36:0). Within this mass range, 13 DAG having 0 to 3 DB occurred (**Table 2**). In addition, other five DAGs (DAG 32:1, DAG 32:2, DAG 34:2, DAG 36:0, and DAG 36:3) were fragmented for confirming their identities (**Supplementary Figures S4D–H**). Based on their fragmentation these DAGs were tentatively assigned as 16:1/16:0; 16:1/16:1; 16:1/18:1; 18:0/18:0; 18:2/16:1, respectively.

**Table 2:** Most abundant DAG molecular ions detected in *X. fastidiosa* subsp. *pauca* strain De Donno (abundance cutoff at 10^3^ counts relative intensity).

	DB:0		DB:1		DB:2		DB:3	
	[M+NH_4_]^+^	RT	[M+NH_4_]^+^	RT	[M+NH_4_]^+^	RT	[M+NH_4_]^+^	RT
DAG 30					554.4784	9.4		
DAG 31					568.4941	10.8		
DAG 32	586.4724	18.4	**584.5202**	**17.8**	**582.5319**	**17.3**		
DAG 33			598.5411	14.3	596.5254	13.2		
DAG 34					**610.5642**	**18**		
DAG 35					624.5567	14.8		
DAG 36	**642.5975**	**19.4**	640.5881	16.2	638.6031	18.8	**636.5786**	**18.2**

*MS/MS experiments have been performed on the m/z highlighted in bold font.*

#### Monoacylglycerides

The LC-TOF analysis of [M+H]^+^ ions of the synthetic MAG 16:0 (*m/z* 331.2772) indicated C_19_H_38_O_4_ as elemental composition. In **Supplementary Figure S5A**, the EIC of m/z 331.2772 showed in XfCFBP8402 MAG isobaric with the standard at comparable RT (6.3 min). As suggested by Han (2016), MAG 16:0 lost 1 H_2_O (m/z 313.2) and RCOOH for the acyl chain (*m/z* 257.2) (**Supplementary Figure S5B**). This fragmentation pattern was present also in XfCFBP8402 (**Supplementary Figure S5C**). Within our samples, MAG were eluted between 2.7 ± 0.04 and 16.0 ± 0.05 min with [M+H]^+^ ions ranging from *m/z* 323.4182 ± 0.0007 (C_17_H_34_O_4_, MAG 14:1) to *m/z* 387.6172 ± 0.0004 (C_23_H_46_O_4_, MAG 20:0). Within this mass range, 15 MAG having 0 to 4 DB occurred (**Table 3**). Most of MAGs were detected as hydrogen adducts with the exception of MAG 14:0, MAG 14:1, MAG 17:0, MAG 19:0 that were detected as sodiated monoacylglycerides. In addition to MAG 16:0 (see above), other two MAG (MAG 18:0, MAG 20:3) within this list (**Table 3**) were fragmented for confirming their identities (**Supplementary Figures S5D,E**).

**Table 3:** Most abundant MAG molecular ions detected in *X. fastidiosa* subsp. *pauca* strain De Donno (abundance cutoff at 10^3^ counts relative intensity).

	DB:0		DB:1		DB:2		DB:3		DB:4	
	[M+H]^+^	RT	[M+H]^+^	RT	[M+H]^+^	RT	[M+H]^+^	RT	[M+H]^+^	RT
MAG 14	325.4392 [M+Na]^+^	3.2	323.4182 [M+Na]^+^	2.7						
MAG 16	**331.2843**	**6.3**								
MAG 17	367.2819 [M+Na]^+^	7.6								
MAG 18	**359.3156**	**10.6**	357.2999	10.2	355.2843	7.9	353.2686	6.4	351.2531	3.3
MAG 19	395.3132 [M+Na]^+^	14.9								
MAG 20	387.6172	16.0	385.5972	15.9	383.5872	15.0	**381.2999**	**14.0**	379.5672	13.4

*MS/MS experiments have been performed on the m/z highlighted in bold font.*

### Identification of Glycerophospholipids

#### Glycerophospholipids

Authentic 1,2-dipalmitoyl-*sn*-glycero-3-phospho-*rac*-(1-glycerol) (PG 16:0/16:0) was analyzed with LC-TOF-MS under conditions specified in the Section “Materials and Methods.” The analysis showed that [M−H]^−^ ions were generated prevalently with ^−^ESI source suggesting an elemental composition of C_38_H_75_O_10_P for the ion with *m/z* 721.5025. The EIC at *m/z* 721.5025 in XfCFBP8402 showed that PG isobaric with the standard was present at comparable RT (14.9 min) (**Supplementary Figure S6A**). Fragment ions in the PI scans of PG 16:0/16:0 of the standard and of XfCFBP8402 cell extracts were consistent with the loss of palmitic acid (*m/z* 255.3) (**Supplementary Figures S6B,C**). Identification of each PG was performed on database^1^ and through recursive analysis on LC-TOF spectra searched for exact mass. The [M−H]^−^ ions of PG in XfCFBP8402 ranged from *m/z* 453.2259 ± 0.002 (C_20_H_38_O_9_P, PG 14:1) to *m/z* 775.5495 ± 0.0007 (C_42_H_80_O_10_P, PG 36:1) for a total of 29 PG having 0–2 DB (**Table 4**). Among these, we characterized four PGs (PG 32:2, PG 32:1, PG 34:2, PG 34:1) for confirming their identities (**Supplementary Figures S6D–G**). Based on their fragmentation, these PGs were tentatively assigned as 16:1/16:1; 16:0/16:1; 18:1/16:1; 18:1/16:0, respectively.

**Table 4:** Most abundant PG molecular ions detected in *X. fastidiosa* subsp. *pauca* strain De Donno (abundance cutoff at 10^3^ counts relative intensity).

	DB:0		DB:1		DB:2	
	[M-H]^−^	RT	[M-H]^−^	RT	[M-H]^−^	RT
PG 14			453.2259	6.7		
PG 16	483.2729	7.9	481.2572	7.1		
PG 18			509.2885	8.6		
PG 20	539.3355	9.8	537.3198	9.5		
PG 21	553.3511	10.2	551.2697			
PG 22	567.3668	10.7	565.3511	10.4		
PG 26	637.4086	12.4	635.3930	12.4		
PG 27			649.4086	12.5		
PG 28			663.4243	13.0	661.4379	12.5
PG 29			677.4399	13.4		
PG 30			691.4556	13.8		
PG 31			705.4701	14.2		
PG 32	721.5025	15.0	**719.4869**	14.6	**717.4731**	14.0
PG 33			733.5010	15.1	731.4830	14.6
PG 34			**747.5229**	15.4	**745.5039**	14.8
PG 35			761.5088	15.8	759.5182	15.2
PG 36			775.5495	16.3	773.5331	15.7

*MS/MS experiments have been performed on the m/z highlighted in bold font.*

#### Choline Glycerophospholipids

Mass accuracy and isotope distribution of the [M+NH_4_]^+^ ions of the authentic 1,2-dinonadecanoyl-sn-glycero-3-phosphocholine (PC 19:0/19:0) indicated that the elemental composition was C_46_H_96_N_2_O_8_P for the ion with *m/z* 836.6899 (**Supplementary Figure S7A**). Fragment ions (*m/z* 356.0) in the PI scans of PC 19:0/19:0 of the standard and XfCFBP8402 (**Supplementary Figures S7B,C**) were consistent with the loss of phosphocholine (*m/z* 183) and a non-adecanoic acid chain (*m/z* 298) in agreement with Han (2016). The precursor ion analysis in positive ion mode searching for the [M+H]^+^ ion of phosphocholine with *m/z* 184.2 showed that PC was eluted between 14.9 ± 0.02 and 21.5 ± 0.05 min. The [M+H]^+^ ions of PC in XfCFBP8402 ranged from *m/z* 650.4762 ± 0.001 (C_34_H_68_NO_8_P, PC 26:0) to *m/z* 810.6014 ± 0.0004 (C_46_H_84_NO_8_P, PC 38:4) for a total of 20 PCs (**Table 5**). Two PCs (PC 34:1, PC 34:2) within this list (**Table 5**) were fragmented for confirming their identities (**Supplementary Figures S7D,E**) and tentatively assigned as 16:0/18:1; 16:0/18:2, respectively.

**Table 5:** Most abundant PC molecular ions detected in *X. fastidiosa* subsp. *pauca* strain De Donno (abundance cutoff at 10^3^ counts relative intensity).

	DB:0		DB:1		DB:2		DB:3		DB:4		DB:5	
	[M+H]^+^	RT	[M+H]^+^	RT	[M+H]^+^	RT	[M+H]^+^	RT	[M+H]^+^	RT	[M+H]^+^	RT
PC 26	650.4762	14.9										
PC 27			662.4762	15.8								
PC 28			676.4918	16.3								
PC 29	692.5231	16.7	690.5075	16.7	688.4918	16.1						
PC 30	706.5388	17.3	704.5231	17.2	702.5075	16.5						
PC 31	720.5544	17.6	718.5388	17.5	716.5231	16.9						
PC 32	734.5701	19.5	732.5544	19.3								
PC 34			**760.5857**	**18.8**	**758.5701**	**18.7**						
PC 36					786.6014	20.1			782.5701	19.7		
PC 38									810.6014	21.5	808.5857	21.3

*MS/MS experiments have been performed on the m/z highlighted in bold font.*

#### Ethanolamine Glycerophospholipids

In our samples, the [M+H]^+^ ion of 2-diheptadecanoyl-sn-glycero-3-phosphoethanolamine (PE 34:0; *m/z* 720.5538; C_39_H_79_NO_8_P) was present at a comparable RT (17.8 min) (**Supplementary Figure S8A**). For structural elucidation, under positive ion mode we detected fragment ions in the PI scans of the standard PE 17:0/17:0 as well as in XfCFBP8402 cell extracts consistent with the abundant NL of phosphoryl ethanolamine ion (*m/z* 141.1) (**Supplementary Figures S8B,C**). The search of NL at *m/z* 141.1 showed that 7 PEs were eluted between 15.4 ± 0.01 and 18.4 ± 0.02 min in XfCFBP8402 cell extracts. Their [M+H]^+^ ions ranged from *m/z* 662.5119 ± 0.0003 (C_36_H_73_NO_7_P, PE O31:1/P31:0) to *m/z* 732.5538 ± 0.0007 (C_40_H_78_NO_8_P, PE 35:1; **Table 6**). Six out of seven PEs (PE O31:1/P31:0, PE O32:1/P32:0, PE O33:1/P33:0, PE O34:2/P34:1, PE 34:2, PE 35:1) were fragmented for confirming their identities (**Supplementary Figures S8D–I**). The “O-” prefix is used to indicate the presence of an alkyl ether substituent, whereas the “P-” prefix is used for the 1Z-alkenyl ether substituent. It was not possible to deduce PE identities since fragmentation did not provide insights in FA composition (loss of ethanolamine).

**Table 6:** Most abundant PE molecular ions detected in *X. fastidiosa* subsp. *pauca* strain De Donno (abundance cutoff at 10^3^ counts relative intensity).

	DB:0		DB:1		DB:2	
	[M+H]^+^	RT	[M+H]^+^	RT	[M+H]^+^	RT
PE O31			**662.5119**	15.4		
PE O32			**676.5276**	16.6		
PE O33		1	**690.5432**	16.5		
PE O34					**702.5432**	16.9
PE 34	720.5538	17.8			**716.5225**	17.4
PE 35			**732.5538**	18.4		

*MS/MS experiments have been performed on the m/z highlighted in bold font.*

### Identification of Phosphorus-Free Lipids

#### Bacteriohopanepolyols (BHPs)

We analyzed our samples for the bacteriohopane-32,33,34,35-tetrol (BHT; m/z 546.4648) that represents the common structural motif among every BHPs. In agreement with previous reports (Spencer-Jones et al., 2015), under positive ion mode, it is possible to detect fragment ions in the PI scans (**Supplementary Figures S9A–C**) of bacteriohopane-32,33,34,35-tetrol, in the standard as well as in our samples, consistent with the cleavage of the ring-C (i.e., loss of *m/z* 192 from the side chain; Talbot et al., 2003). In our samples, we found 8 BHPs that were eluted between 15.7 ± 0.03 and 19.7 ± 0.01 min and their [M+H]^+^ ions ranged from *m/z* 547.4722 ± 0.0004 (C_35_H_62_O_4_, bacteriohopane-32,33,34,35-tetrol) to *m/z* 758.7022 ± 0.0005 (C_49_H_91_NO_4_, N-hexadecanoyl-35-aminobacteriohopane-32,33,34-triol) (**Table 7**). Bacteriohopane-32,33,34,35-tetrol found in XfCFBP8402 cell extracts was fragmented for confirming its identity against the standard (**Supplementary Figure S9C**).

**Table 7:** Most abundant BHP molecular ions detected in *X. fastidiosa* subsp. *pauca* strain De Donno (abundance cutoff at 10^3^ counts relative intensity).

Compound name	[M+H]^+^	Formula	RT
methyl-adenosylhopane	676.5271	C_41_H_65_N_5_O_3_	15.7
**bacteriohopane-32,33,34,35-tetrol**	**547.4722**	**C_35_H_62_O_4_**	**16.8**
bacteriohopane-31,32,33,34,35-pentol	563.4671	C_35_H_62_O_5_	17.6
bacteriohopane-31,32,33,34-tetrol-35-cyclitol	724.5285	C_41_H_73_NO_9_	17.9
bacteriohopane-,32,33,34, 35-tetrol-glucosamine	708.5410	C_41_H_73_NO_8_	18.2
bacteriohopane-31,32,33,34,35-hexol	579.4665	C_35_H_62_O_6_	18.5
N-hexadecanoyl-35-aminobacteriohopane-32,33,34-triol	758.7022	C_49_H_91_NO_4_	18.5
bacteriohopane-,32,33,34-triol-35-cyclitolguanine	750.5628	C_42_H_75_N_3_O_8_	19.7

*MS/MS experiments have been performed on the m/z highlighted in bold font.*

#### Ornithine-Containing Lipids (OL)

Authentic OL1 15:0-OH/19:0 cyclo was analyzed and its [M+H]^+^ ion with *m/z* 651.5186 indicated an elemental composition C_39_H_74_N_2_O_5_. Both standard and corresponding sample OL (in XfCFBP8402 cell extracts) were subjected to MS/MS fragmentation. In agreement with previous reports (Geske et al., 2013; Escobedo-Hinojosa et al., 2015), under positive ion mode, it is possible to detect fragment ions in the PI scans (**Supplementary Figures S10A,B**) of the standard OL as well as in our samples. PI scans are consistent with the abundant loss of ornithine (–H_2_O) (*m/z* 115.0866) and the NL of the 19:0 cyclo FA resulting in a fragment ion at *m/z* 369.3081. This OL species was not the most abundant among the OLs of XfCFBP8402. To extend the spectrum of OL within our samples, we performed a precursor ion analysis in positive ion mode searching for the [M+H]^+^ ion of orn–H_2_O with *m/z* 115.1 and of orn+OH with *m/z* 131.0764. The latter search did not provide any significant results; precursor ion search in positive ion mode of *m/z* 115.1 showed that OL were eluted between 17.7 ± 0.01 and 19.5 ± 0.02 min. Identification of each OL was performed on available references (Zhang et al., 2009; Geske et al., 2013; Escobedo-Hinojosa et al., 2015) and through recursive analysis on LC-TOF spectra searched for exact masses. The [M+H]^+^ ions of 11 OLs found in the XfCFBP8402 cell extracts ranged from *m/z* 651.4760 ± 0.0003 (C_39_H_74_N_2_O_5_, OL 34:0) to *m/z* 912.2092 ± 0.0007 (C_57_H_78_NO_8_, OL 52:0; **Table 8**). Two OLs (OL 34:0, OL 36:0) within this list were fragmented for confirming their identities (**Supplementary Figures S10C,D**). Based on their fragmentation and on reference available, these OLs were tentatively assigned as OL1 (15:0/19:0 cyclo) and 17:0/19:0.

**Table 8:** Most abundant OL molecular ions detected in *X. fastidiosa* subsp. *pauca* strain De Donno (abundance cutoff at 10^3^ counts relative intensity).

Compound name	[M+H]^+^	Formula	RT
OL 34:0	**651.5186**	C_39_H_75_N_2_O_5_	17.7
OL 36:0	**680.4838**	C_41_H_80_N_2_O_5_	17.9
OL 38:0	708.5410	C_43_H_84_N_2_O_5_	18.0
OL 42:3	760.5840	C_47_H_88_N_2_O_5_	18.0
OL 40:0	737.5628	C_45_H_89_N_2_O_5_	18.3
OL 42:0	766.5850	C_47_H_94_N_2_O_5_	18.4
OL 44:0	795.6155	C_49_H_99_N_2_O_5_	18.5
OL 46:0	824.6258	C_51_H_104_N_2_O_5_	18.8
OL 48:0	854.1474	C_53_H_109_N_2_O_5_	18.9
OL 50:0	883.1660	C_55_H_114_N_2_O_5_	19.2
OL 52:0	912.2092	C_57_H_119_N_2_O_5_	19.5

*MS/MS experiments have been performed on the m/z highlighted in bold font.*

### Analysis of Specific Lipid Groups

**FIGURE 1:**
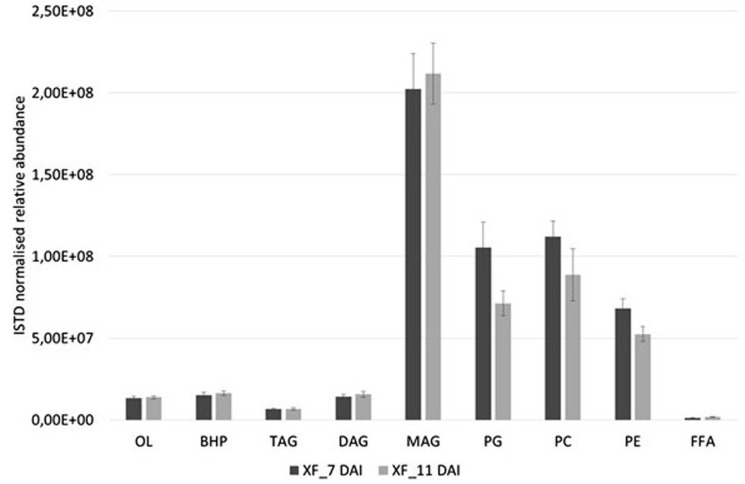
Relative abundances of diverse lipid class extracted from *X. fastidiosa* CoDiRO strain CFBP8402 at different time intervals (7–11 DAI). Bars represent the sum of the ISTD-normalized area of single compounds within each category of lipids. Results are the mean ( ± SE) of 10 independent experiments (*n* = 6 in each experiments).

Basing on the lipid entities found above, we measured the relative abundance of several MAG, DAG, TAG, OL, BHP, PG, PC, and PE in the cell extracts of *X. fastidiosa* CoDiRO strain CFBP8402 at different times of growth (7–11 DAI) (**Supplementary Figure S1A,B** and **Supplementary Table S4**). Rough indications provided that MAG and PL fractions prevailed into bacterial cells even if BHPs represented either a significant portion of this list. Within each lipid category, some specific compounds emerged as prevalent; for instance MAG (16:0), (18:0), and (20:3) within monoacylglycerides; DAG 36:5 (18:2/18:3) within diacyliglycerides; TAG 49:0 (15:0/16:0/18:0) and 52:2 (16:0/18:1/18:1) within triacylglycerides; OL1 (15:0/19:0 cyclo) within OL; BHT within BHPs; and PG 32:2 (16:1/16:1) and 34:2 (18:1/16:1), PE 36:3 (18:1/18:2), and PC 34:2 (16:1/18:1) within PL (**Supplementary Table S4**). The **Figure 1** shows that the relative abundance of lipid entities differed significantly among 7 and 11 DAI for the sole PG (*p* < 0.01) and PE (*p* < 0.05).

### Targeted Analysis of FFA and Oxylipins

**FIGURE 2:**
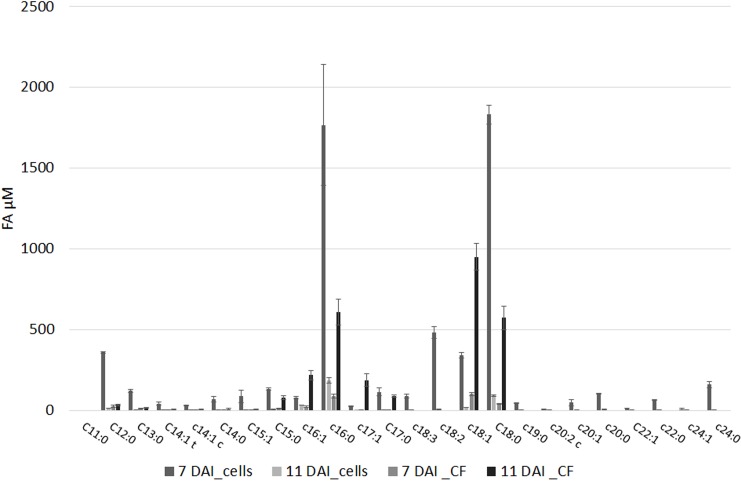
Free fatty acids (FFAs) profile of (i) pelleted cells of *X. fastidiosa* CoDiRO CFBP8402 at different time intervals (7 and 11 DAI) and (ii) culture filtrate harvested at 11 DAI. Results are the mean ( ± SE) of 10 independent experiments (*n* = 6 in each experiments).

The FFA profile shows the presence of multiple compounds (**Figure 2** and **Supplementary Table S5**): main FFA was stearic (C18:0), followed per abundance by oleic (C18:1), palmitic acid (C16:0), linoleic (C18:2), and heptadecenoic acid (C17:1). Unsaturated FA (UFA) and polyunsaturated fatty acids (PUFA) such as myristic, palmitoleic, and linolenic acid (C14:0, C16:1, and C18:3, respectively) even if in minor amount were present in our samples. The abundance of the different FFA was similar in cell and in culture filtrate, with the sole exceptions of palmitoleic (XfDSF2) and oleic acid which are more abundant at 11 DAI in the culture filtrate than in the cell extracts (**Figure 2**). In our samples, we also detected XfDSF1 in *trans* and *cis* forms (C14:1 t and c, respectively; **Figure 2**). Every FFA, significantly (*p* < 0.001) changed during bacterial growth.

*Xylella fastidiosa* produced several oxylipins deriving from oleic, linoleic and linolenic acid (**Table 9** and **Supplementary Table S6**). Some oxylipins are produced at significant, micromolar amount, in the bacterial cells as well as in the culture filtrate. In **Table 9**, we showed the sole oxylipins present in significant amount; a more comprehensive view of the complete profile is reported in **Supplementary Table S6**. Among the most abundant compound, the products of oxylipins derived from oleic and linoleic acid were dominant. Notably, 10-HOME, 10HpOME (derived from oleic acid), and 12,13-diHOME, 9,10-diHOME, 12,13-epOME, 9,10-epOME (derived from linoleic acid) even with different intra/extra-cell distribution, prevailed over the others oxylipins. Intriguingly, 7,10-diHOME (the major product of 7,10-diol synthase; Estupiñán et al., 2014) shows values < LOQ under *in vitro* conditions. Other, less represented, oxylipins such as 13-HODE 9-HODE, 8,13-diHODE, 13HOTrE, and methyl jasmonic acid (JA) were found in the cells as well as in the culture filtrate.

**Table 9:** Quantification of a defined set of 11 oxylipins into *X. fastidiosa* CoDiRO CFBP8402 cells and culture filtrate (CF) at 7 and 11 days after inoculation (DAI).

	Oxylipins μM			
	7 DAI_cells	11 DAI_cells	7 DAI_CF	11 DAI_CF
**10-HOME**	381.5 ± 23.2	181.6 ± 12.2	0.4 ± 0.05	1670.1 ± 18.4
**10-HpOME**	0.02 ± 0.005	0.04 ± 0.01	7.1 ± 1.2	10.9 ± 1.2
**12,13 di HOME**	< LOQ	< LOQ	2.2 ± 0.4	48.1 ± 4.2
**9,10-diHOME**	< LOQ	< LOQ	0.2 ± 0.1	0.7 ± 0.1
**12,13-epOME**	0.01 ± 0.004	0.01 ± 0.002	0.05 ± 0.01	226.9 ± 35.2
**9,10-epOME**	0.003 ± 0.001	0.002 ± 0.001	0.2 ± 0.1	457.3 ± 50.2
**13-HODE**	0.005 ± 0.001	0.005 ± 0.001	0.1 ± 0.05	0.09 ± 0.01
**9-HODE**	0.002 ± 0.0002	0.002 ± 0.0005	0.15 ± 0.05	75.5 ± 8.2
**8,13-diHODE**	< LOQ	< LOQ	0.1 ± 0.05	48.2 ± 2.1
**13HOTrE**	< LOQ	< LOQ	0.2 ± 0.1	0.003 ± 0.001
**Methyl jasmonic acid**	0.003 ± 0.001	0.001 ± 0.0002	3.1 ± 0.4	0.03 ± 0.002

*Results are the mean (± SE) of 10 independent experiments (*n* = 6 in each experiments).*

### Infection of a Model Plant: *Nicotiana tabacum*

**FIGURE 3:**
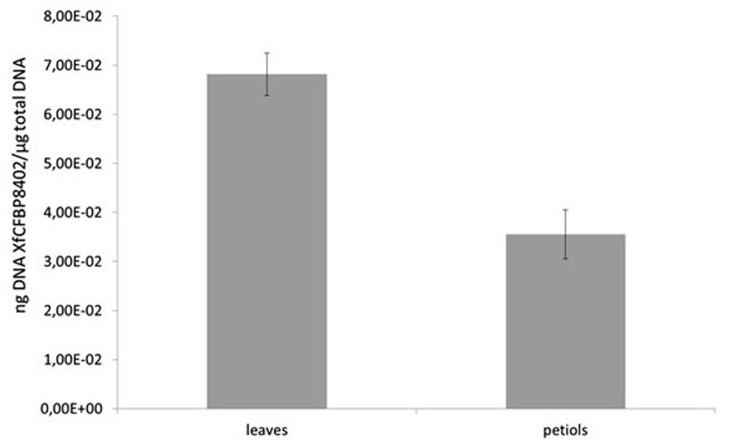
qPCR of XfCFBP8402 DNA in the laminar part of the leaf (named leaves) and in the petioles and central vein (named petioles) at 30 DAI. Results represent the average of ng DNA of XfCFBP8402/μg of plant total DNA ± SE of three independent experiments (*n* = 15, in each experiment).

The presence of XfCFBP8402 in experimentally inoculated tobacco tissues was confirmed through real-time qPCR. Its distribution differed significantly (*p* < 0.001) among petioles and leaves (**Figure 3**); the pathogen was not detected in the mock plants. In particular, the DNA of XfCFBP8402 was higher in the leaves than in the petioles of Xf-infected plants (**Figure 3**).

**FIGURE 4:**
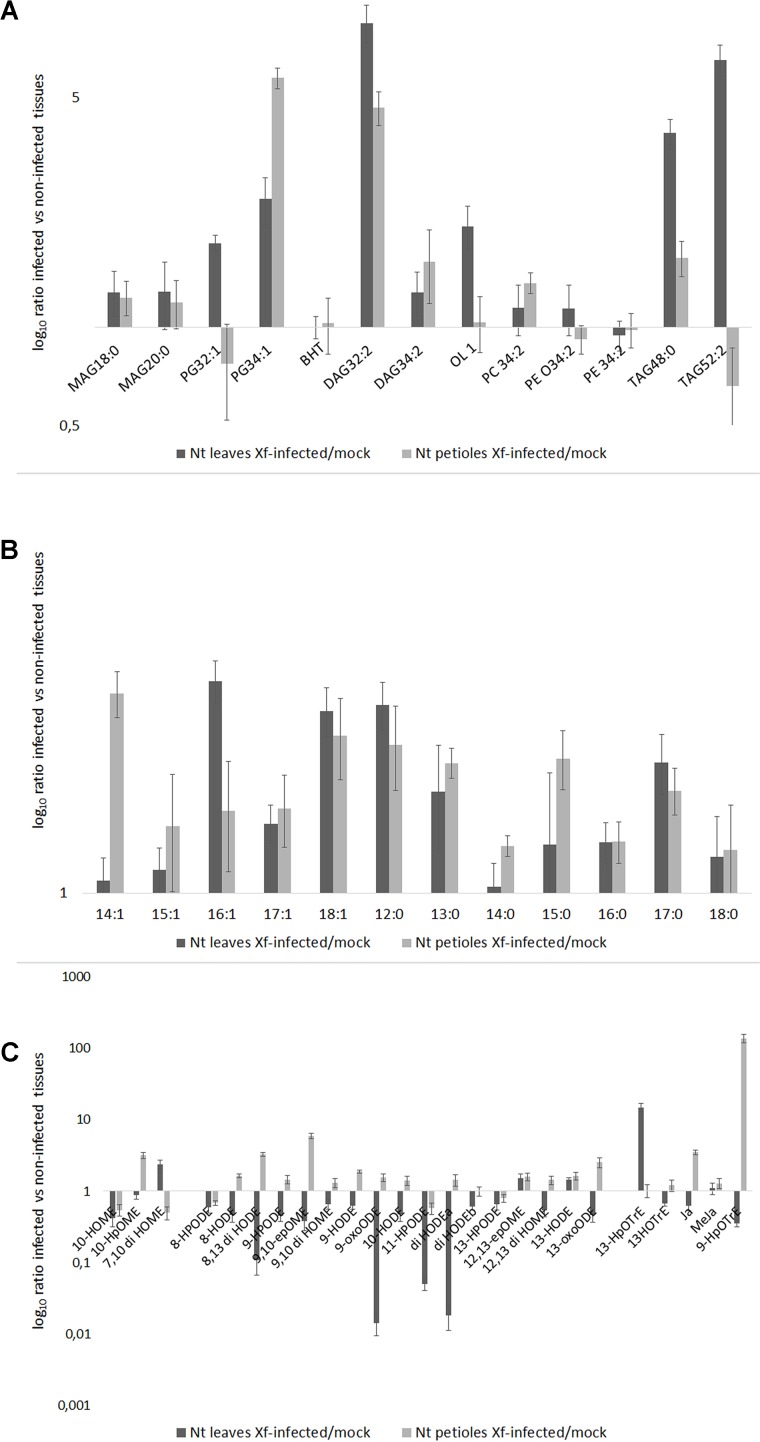
**(A)** Lipid entities, **(B)** Free fatty acids (FFAs), and **(C)** Oxylipins in tobacco petioles and leaves in mock and Xf-infected samples at 30 DAI. Results represent the average of log10 ratio of the fold change in the abundance of the different lipid compounds present in the infected samples compared to mock ± SE of three independent experiments (*n* = 15, in each experiment).

The lipid profile of the leaves and petioles of the Xf-infected and mock plants was studied. The analysis was focused on those lipid entities that were most abundant in the previous *in vitro* screening of XfCFBP8402 (see above; **Figures 4A–C**). The results were reported as the amount of lipid entities in the XfCFBP8402-infected plants compared to uninfected ones (mock). Raw data (relative abundance of each lipid entities into plant tissues) are presented in **Supplementary Tables S7–S9**.

All the lipids entities analyzed were more abundant in the Xf-infected samples than in the mock; PG 32:1 (16:1/16:0), TAG 52:2 (16:0/18:1/18:1), BHT, and PEs represent the sole exceptions (**Figure 4**). In particular, PG 32:1 and TAG 52:2 together with OL1 were more abundant in the leaves compared to the petioles (*p* < 0.001 for each of the three compounds; **Figure 4A** and **Supplementary Table S7**).

After 30 DAI, all FFA were more abundant in Xf-infected samples than in the mock, in the leaves as well in the petioles (**Figure 4B**). In particular, C14:1 (XfDSF1) was particularly abundant in the petioles while the C16:1 (XfDSF2) in the leaves. Other potential DSF emerged within this analysis: C15:1, C17:1 and – most notably – C18:1 (confirming *in vitro* data – **Figure 2**).

The **Figure 4C** shows the oxylipins derived by oxidation of oleic, linoleic and linolenic acid, respectively. In the **Figure 4C**, the distribution of the oxylipins appeared consistently different between the petioles and the leaves; notably, mostly upregulated in the petiole and downregulated in the leaves. In our experimental conditions, 10-HpOME was more abundant in the petioles than in the leaves; conversely, 7,10-diHOME is more abundant in the leaves than in the petioles. The oxylipins derived by linoleic acid are more abundant in the petioles than in the leaves, made exception 12(13)-epOME and 13-HODE. In the petioles of the Xf-infected plants, 9-HpOTrE is the prevailing oxylipin derived from linolenic acid together with JA, whereas 13-HpOTRE is more abundant in the leaves.

## Discussion

Within the last decade, an increasing number of studies identified lipids in phytopathogenic bacteria and described their role in the interaction with the hosts. Lipids are essential constituents of the cells providing different functions ranging from structural to energy storage and signal mediators (Lim et al., 2017). *Escherichia coli* has been the standard model to study bacterial lipids for years; very recently, Sohlenkamp and Geiger (2016) reported the diversity of membrane lipids in a multitude of eubacteria. This “burst” in lipid surveys in bacteria relies also on the stepping up of analytical methods essentially based on coupling chromatography with mass spectrometry.

In the present study, we have addressed an analytical method integrating exact mass data (LC-TOF) with mass fragmentation (LC-MS/MS) to detect multiple classes of lipids in the XfCFBP8402 associate with severe epidemics in centenarians olive trees in Southern Italy. Currently, a holistic approach is under way for containing this outbreak. Studying the bacterial lipid diversity could aid the individuation of the lipid entities potentially involved in the interaction with host. Through this method, we succeed in evaluating the presence of different TAG, DAG, MAG, PC, PE, OL, OH, BHPs, FFA, and oxylipins in this pathogen. From what we know at present, this is the first report concerning complex lipids, FFA, and oxylipins in CFBP8402. We approached a method providing information on several intra- and extra-cellular lipids of XfCFBP8402 grown under *in vitro* conditions as well as *in planta*.

Different authors (Sohlenkamp and Geiger, 2016; Rowlett et al., 2017) report that nutrient availability, accumulation of products of metabolism, pH variation, oxygen levels, biofilm formation, and surface adhesion significantly affect the lipid composition of bacteria grown under *in vitro* conditions. In our study, we analyze the lipid composition of the bacterial culture at 7 and 11 DAI to check if lipid entities composition change during the growth phase as reported by Feil and Purcell (2001). Notably, within this time frame (7–11 DAI) in our growing conditions, XfCFBP8402 shifts from a planktonic behavior to form the characteristic cell ring at the air-liquid interface. By combining TOF and MS/MS analysis, we succeed in identifying different entities within each lipid class: 18 TAGs, 13 DAGs, 15 MAGs, 29 PGs, 20 PCs, 7 PEs, 8 BHPs, 11 OLs, 23 FFA, and 24 oxylipins, respectively. The relative abundance of MAG, DAG, TAG, OL, BHP, PG, PC, and PE indicated that MAG and PL fractions prevailed in bacterial cells. With current data, we can suggest that lipid changes occurring between 7 and 11 DAI may influence the cells physicochemical properties like membrane-protein topology, inner and outer membrane transport, modulate the interaction among bacterial cells (e.g., quorum sensing), shift from planktonic growth to biofilm formation (Bakholdina et al., 2004; Zhang and Rock, 2008; Bisbiroulas et al., 2011).

The FFA fraction represents either an important reservoir of signal molecules *per se* or as precursor readily converted to oxylipins (Yaeno et al., 2004; Siebers et al., 2016). Within the class of FFA, DSFs are gaining *momentum*. DSF families have been described in different bacterial pathogens; these (mono)unsaturated FAs (UFA) are involved in interspecies and inter-kingdom signaling and recognition. In *Xylella* and *Xanthomonas*, different authors indicate that DSF signals are fine-tuned during interaction with the host plants since they are recognized as elicitors, thus triggering innate immune response in plants (Torres et al., 2007; Chatterjee et al., 2008a; Kakkar et al., 2015). *X. fastidiosa* utilizes one or more of such signal molecules to shape its *lifestyle* depending on cell density (Ionescu et al., 2016; Dow, 2017). In *X.* *fastidiosa*, RpfF – a bifunctional crotonase with both dehydratase and thioesterase activities – produces a mixture of DSF species (from C14:1 to C19:1) during the invasion of the xylem of host plants (Wang et al., 2004; Ionescu et al., 2016). *X. fastidiosa* DSF-deficient mutants are more virulent but less capable of colonizing the insect vector and infecting healthy plants (Newman et al., 2004; Chatterjee et al., 2008b). Currently, at least two DSF, XfDSF1 (C14:1) and XfDSF2 (C16:1) were thoroughly investigated; XfDSF2 is apparently more active as signaling molecule compared to XfDSF1 (Ionescu et al., 2016).

In this paper, we show that in culture filtrate (and in cell extracts as well) of XfCFBP8402 strain, XfDSF2 is more abundant compared to XfDSF1 that, in turn, is more abundant at 7 DAI than 11 DAI of growth. In general, this strain appears competent in producing several UFA (up to C24:1) at least as cell components (FA in complex lipids); indeed, oleic (C18:1) and palmitic acid (C16:1) are the main UFA in FFA fraction. Potentially, other XfDSFs could have a role in different aspects of XfCFBP8402 lifestyle; in fact, both C17:1 and C18:1 accumulate at significant levels in the culture filtrate at 11 DAI. It is suggested elsewhere that different UFAs can have different activities in other strains of *X. fastidiosa*: from quorum sensing to toxicity (Ionescu et al., 2016).

Moreover, FFA can function as substrates to form oxylipins. The biosynthesis of oxylipins occurs both constitutively and consequently to abiotic and biotic stresses. Oxylipins modulate several processes in prokaryotic and eukaryotic cells. In mammals, plants, and fungi, the biosynthetic pathways and physiological roles of some oxylipins are almost well defined: e.g., prostaglandins in mammals, jasmonates in plants, hydroxy-acids in fungi (Blèe, 2002; Andreou et al., 2009; Christensen and Kolomiets, 2011; Scala et al., 2014). According to the available literature, oxylipins might derive from LOX as well as DOX activities, although they can also be produced by non-enzymatic chemical oxidation of FAs (Christensen and Kolomiets, 2011; Banthiya et al., 2016; Martinez and Campos-Gomez, 2016). For a long time, oxylipins studies had been limited to eukaryotes (Andreou et al., 2009); nevertheless, the first two prokaryotic LOX sequences were described (Vance et al., 2004) and numerous oxylipins were subsequently characterized; some of these oxylipins regulate the host–pathogen interaction (Porta and Rocha-Sosa, 2001). Recently, in *P. aeruginosa* the cellular function of oxylipins has gained attention. Among these, (10S)-hydroxy-(8E)-octadecenoic acid (10-HOME), and 7S,10S-dihydroxy-(8E)-octadecenoic acid (7,10-diHOME) are depicted as crucial in determining a switch in bacterial lifestyle; namely, these oxylipins are required for biofilm formation when bacteria interacts with host cells (Martinez and Campos-Gomez, 2016). The authors here suggest that the expression of oxylipin forming genes might alter lipid signaling during the interaction with the host, impact biofilm formation, and improve the invasiveness of the bacteria. The simultaneous detection of oxylipins in the cells as well as in the cultural filtrate suggests indeed that although oxygenation may occur inside the cell, oxylipins are transported through the outer membrane and accumulate in the medium (Martinez et al., 2013).

In this paper, we show that different oxylipins deriving from oleic, linoleic, and linolenic acid are detected both in the cells and in the cultural filtrate of *X. fastidiosa* CoDiRO CFBP8402. Among the most abundant compounds, oxylipins derived from oleic and linolenic acid are dominant. Notably, 10HpOME, 10-HOME, and epOMEs even with different intra/extra-cell distribution prevailed over the others oxylipins. Our results confirm previous findings in *P. aeruginosa* reporting that oxylipins mainly accumulate in the extracellular medium and synthesis ascribed to DOX and diol synthase activities (Martinez et al., 2013; Martinez and Campos-Gomez, 2016). Other, less represented, oxylipins such as 13-HODE 9-HODE, 8,13-diHODE, 13HOTrE, and methyl jasmonic acid, whose synthesis is related to LOX enzymes (Christensen and Kolomiets, 2011) are found into the cells as well as in the culture filtrate. Intriguingly, the well-known plant stress hormone (Nomura et al., 2005) – methyl jasmonate – is produced and, overall, secreted into consistent amount. This compound could play a role in modulating plant defenses if produced even during plant infection. The evidence that these oxylipins were more abundant in culture filtrate, suggesting their secretion and pave the way for hypothesizing a role in the infection path of *X. fastidiosa* similar to that played in *P. aeruginosa*.

The long latency period of *X. fastidiosa* for the perennial hosts, such as citrus and olive, makes arduous studying this pathogen *in planta*. Some authors demonstrated that tobacco can be used as model plant for *X. fastidiosa* subsp. *pauca*, although different varieties of this *Solanacea* show different level of infection and symptoms development (Pereira et al., 2017). In our study, *N. tabacum* Petit Havana SR1 artificially infected with XfCFBP8402 strain is used to monitor the bacterial ability to multiply and spread into tobacco and to study the variation of target lipid entities into XfCFBP8402 infected plants compared to uninfected ones.

The tobacco Xf-infected plants show feeble symptoms after 15 DAI: that are, wrinkling of the lower older leaves along the leaf margin. This wrinkling develops across the leaf’s surface in the interveinal spaces resulting in leaf deformation and in leaf early yellowing (**Supplementary Figure S11**). The distribution of XfCFBP8402 is diverse within the analyzed tissues: more abundant into leaves (laminar part) than in the petioles (and central vein) of the samples. This scenario is almost clearly illustrated by the lipid profile of the two parts of the leaf. The lipid entities whose abundance varying dramatically upon bacterial infection may be ascribed to both plant and pathogen *repertoire* with some exception such as the bacterial ornithine lipid OL1 and the hopanoid BHT. Namely, plant change their lipid profile following pathogen attack (Siebers et al., 2016) as well as bacteria reshape their lipids while infecting the host (Torres et al., 2007; Nascimento et al., 2016; Siebers et al., 2016).

Complex lipids (e.g., DAG) as well as FFA (e.g., oleic acid) follow in the petioles and leaves similar trends with some notable exception. For instance, OL1 (OL 15:0-OH/19:0 cyclo) is more abundant in leaf margins; thus, we can here suggest that OL1 may act as Pathogen-Associated Molecular Patterns (PAMP) and trigger plant reactions in this part of the leaf where bacterial load is high (as indicated by qPCR; Vences-Guzman et al., 2013). TAG 52:2 (16:0/18:1/18:1) and PG 32:1 (16:1/16:0) may represent a source of diffusible factors (e.g., C16:1 and 18:1) in presence of lipase activity. *X. fastidiosa* subsp. *fastidiosa* strain Temecula1 (causal agent of the PD in grapevine), secretes the lipase LesA during the infection of the host. LesA accumulates, inversely with bacterial titer, in leaf margins and positively contributes to develop PD symptoms such as leaf scorching and chlorosis in grapevine. Notably, LesA decreases, in a gradient-shaped manner, toward the petiole where *Xylella* formed a biofilm network (Nascimento et al., 2016). According to our evidences, we can suggest that XfCFBP8402 uses lipase and the products of its activity as virulence factors. For instance, starting from PG 32:1 (16:1/16:0), a putative lipase could release C16:1 (XfDSF2) that is actually more abundant in the leaf than in the petiole. Further, **Figure 4B** shows that different FFA are accumulating in the Xf-infected plants and that the C14:1 (XfDSF1) is more abundant in the petioles than in the leaves (**Supplementary Tables S4–S6**) suggesting that the two DSF have a different role during the interaction with the host. We can argue that XfDSF1 is associated with the formation of biofilm that should occur within the petiole but not into the leaf margins.

**FIGURE 5:**
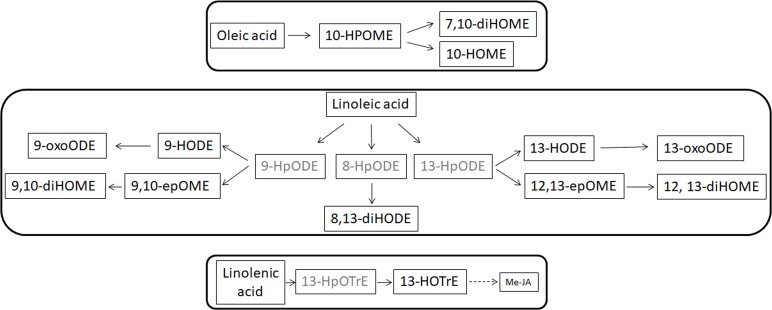
Suggested overview of oxylipin pathway in XfCFBP8402. Black font represents oxylipins that were found in the samples and gray font represents oxylipins that were expected in the samples but not found. Continuous lines represent single step in production of the oxylipins, whereas dotted lines indicate the presence of multiple steps to achieve the compounds indicated in figure.

The FFAs, as indicated elsewhere, provide substrates for the formation of oxylipins. Oxylipins are indicators of plant reactions during stress responses (Blèe, 2002; Christensen and Kolomiets, 2011; Scala et al., 2014; Siebers et al., 2016). The oxylipin profile – whose synthesis overview is reported in **Figure 5** – is completely different in the two leaf parts: enhanced within the petiole while depressed in the leaves. Notable exceptions (i.e., oxylipins enhanced into leaves) are represented by 7,10-diHOME and, the jasmonic acid precursor, 13-HpOTrE. We should here hypothesize that the first may be produced by XfCFBP8402 similarly to *P. aeruginosa* where this oxylipin appears playing a critical role in the initial stages of biofilm formation (Martinez and Campos-Gomez, 2016). 13-HpOTrE levels rise during tobacco infection; JAs, indeed, appear at steady state or feebly decreased. We can here speculate that XfCFBP8402 could interfere actively with the JA-pathway for avoiding plant resistance. Further studies are needed to investigate thoroughly the putative role of these molecules in the interaction with the host plants and finding the enzymes involved in their production.

It emerges a scenario in which the complex lipid profile of XfCFBP8402 – partly elucidated in this study – contributes in several ways to its lifestyle and to its relation with the host. In particular, we can here suggest that other DSFs than the already described ones (i.e., XfDSF1 and 2), as well as other lipid entities characterized in this study (e.g., oxylipins), are worth of further investigation in *X. fastidiosa* pathogenesis. Since the XfCFBP8402 ability to colonize tobacco appears related to the diffusion of lipid factors, it can be argued that lipid entities such as OL1, TAG 52:2, C18:1, and 7,10-diHOME may constitute an arsenal of molecules that actively contribute to plant–pathogen cross-talk.

## Author Contributions

VS performed the conception and design, data interpretation, coordination of contributes, and paper preparation and revision. MR performed the experiment management, data interpretation and elaboration, and paper preparation and revision. MS performed the data management and data acquisition. NP performed the plant infection experiment management and paper revision. VM performed the PCR data acquisition and interpretation. SLu performed the plant infection data acquisition. SLo performed the conception and design, data interpretation, and paper preparation and revision.

## Conflict of Interest Statement

The authors declare that the research was conducted in the absence of any commercial or financial relationships that could be construed as a potential conflict of interest.

## Footnotes

1 www.lipidmaps.org

2 https://metlin.scripps.edu/landing_page.php?pgcontent=mainPage
