# Supplementary material for: Lipid Profile of Xylella fastidiosa Subsp. pauca Associated With the Olive Quick Decline Syndrome
Source: Front Microbiol. 2018 Aug 14;9:1839. doi: 10.3389/fmicb.2018.01839 (PMC6102392; doi:10.3389/fmicb.2018.01839)
Supplement: Supplementary file 2 [file Data_Sheet_2.docx]

# Supplementary Figures 1-11 and Tables 1, 3, 5

A) Positive Ion mode


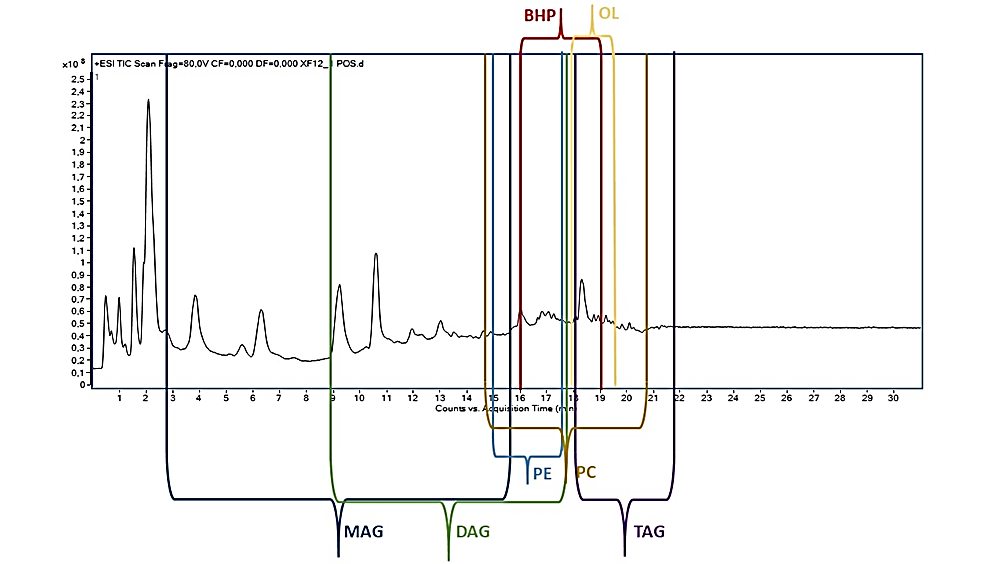

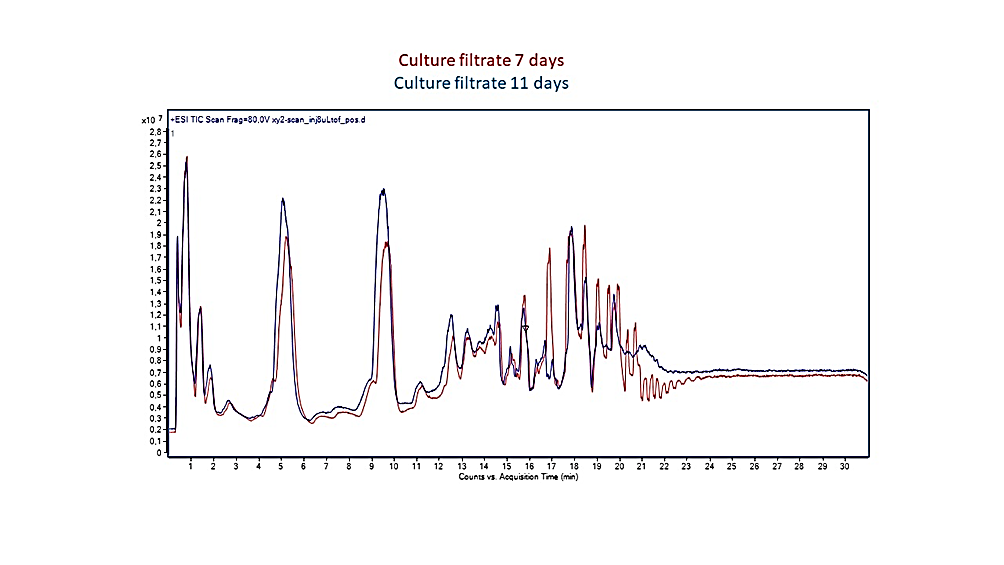

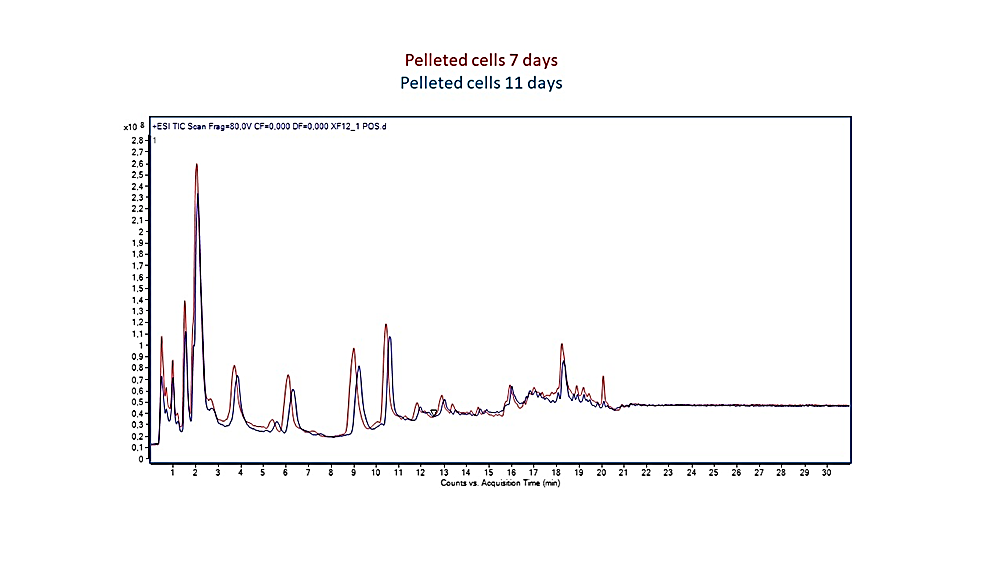


B) Negative ion mode


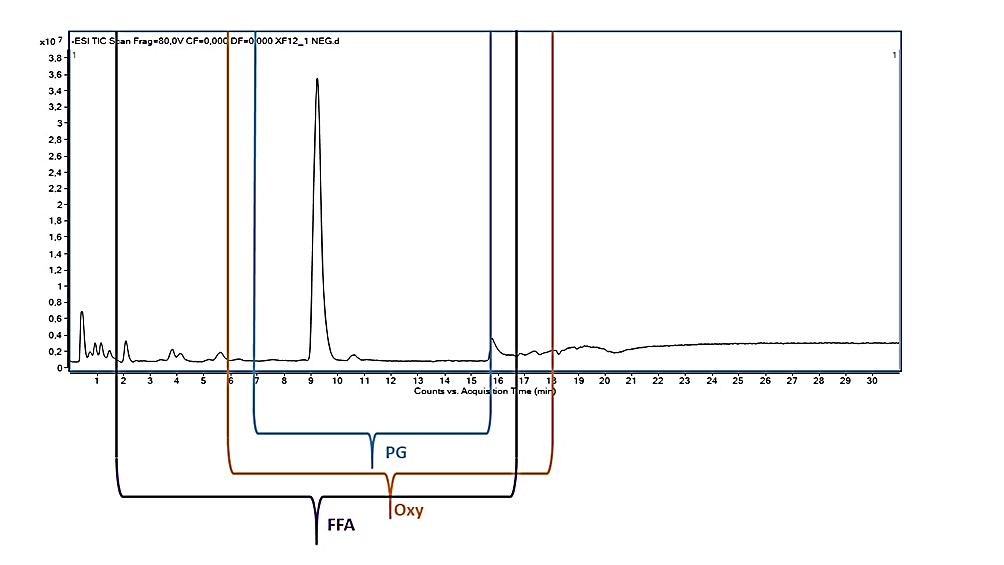

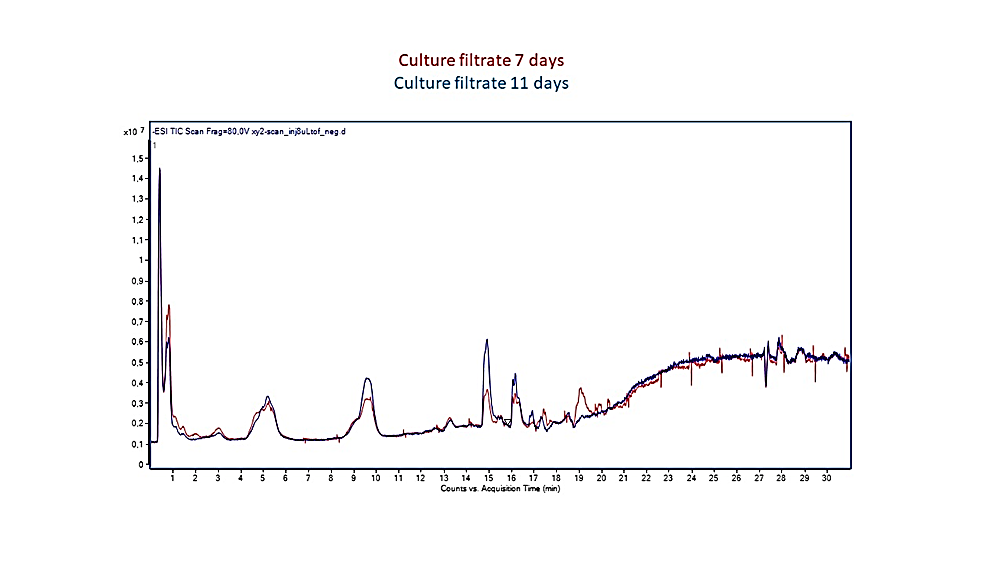


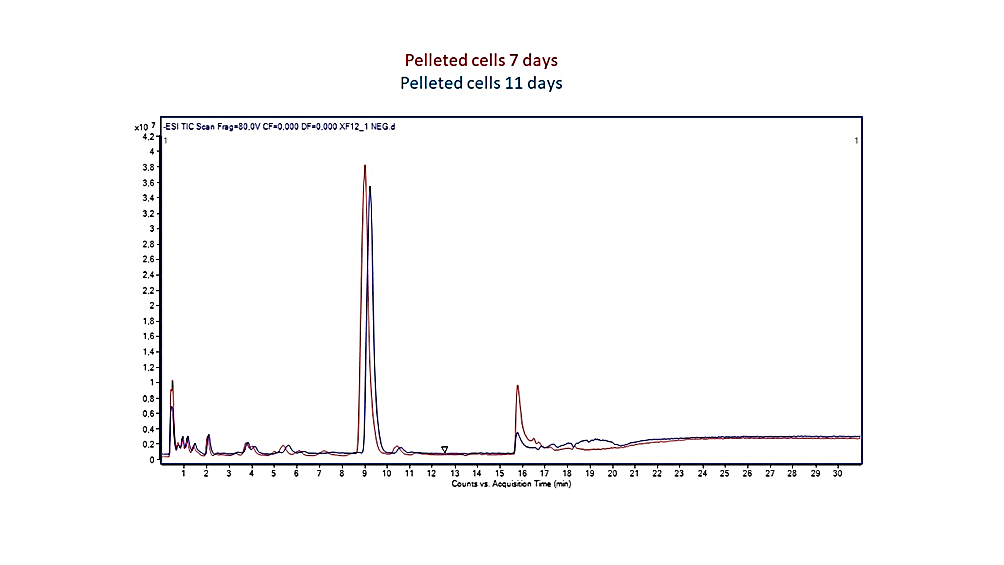


**Supplementary Figure 1 A,B.** TIC chromatogram of TOF mass spectra acquired in positive (A) and negative (B) ion mode of XfCFBP8402 pelleted cells and culture filtrates at 7 and 11 days after inoculation. Rough indications of RT corresponding to each lipid class analyzed are provided between brace.


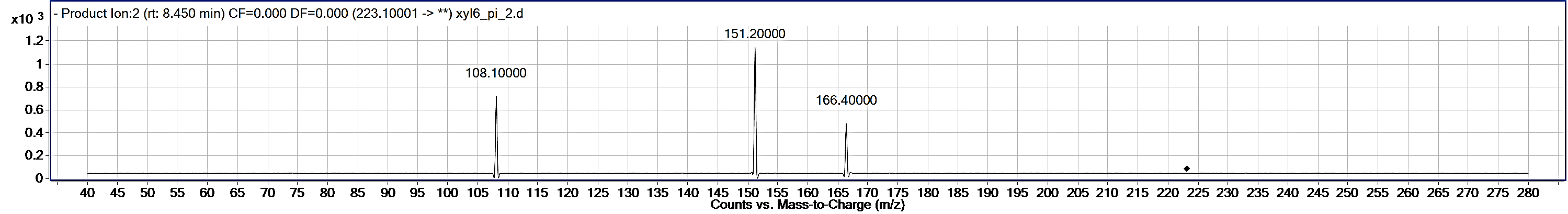


JA


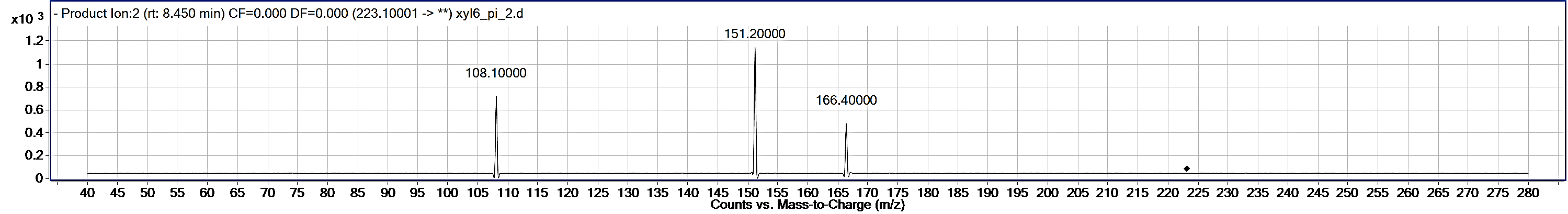


MeJA

**Supplementary Figure 2.** Fragmentations of methyl-jasmonate and jasmonic acid into bacterial cell extracts (Balcke et al. 2012). MRM data were processed using the Mass Hunter Quantitative software (B.07.00 version).

A)

B)


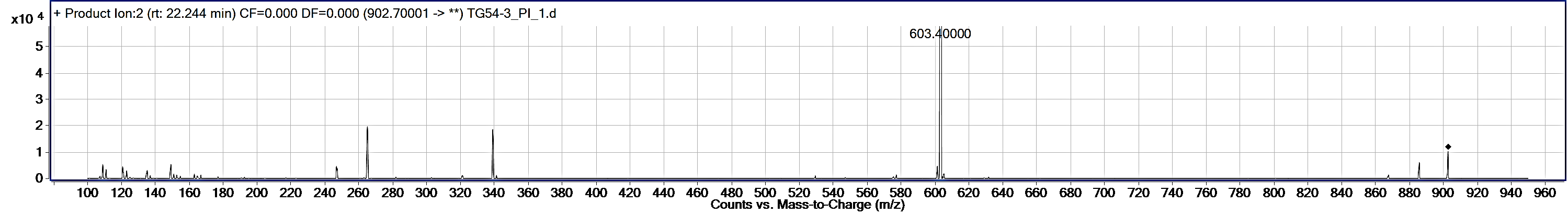


C)
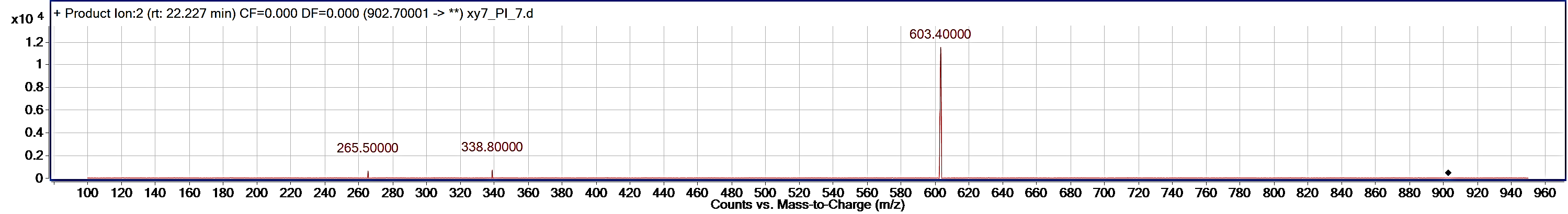


D)
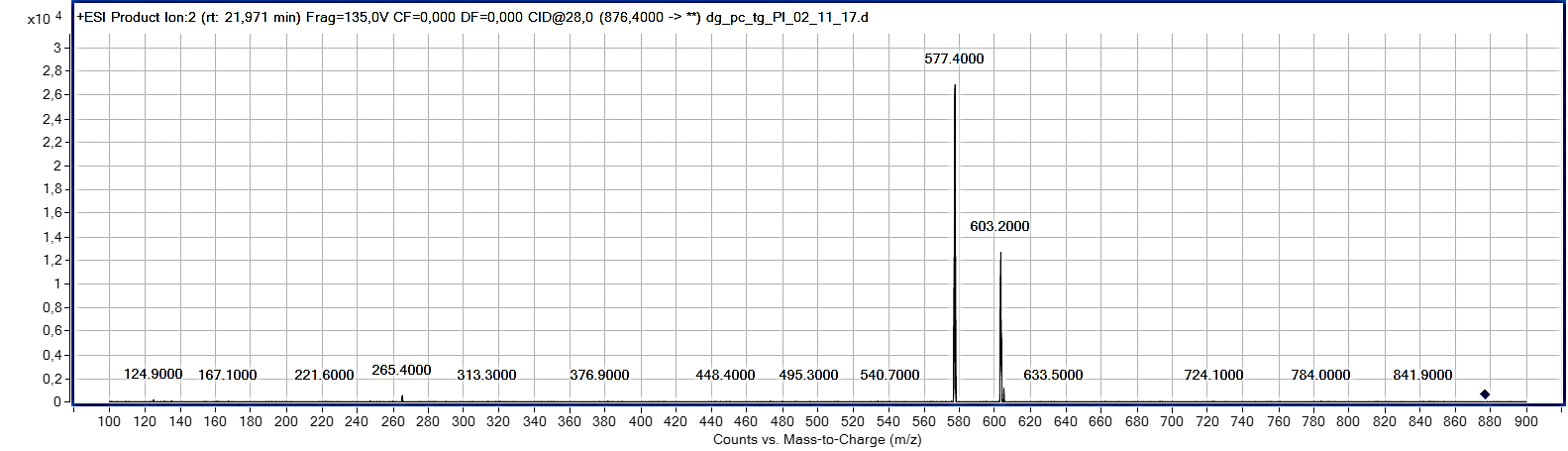


E)
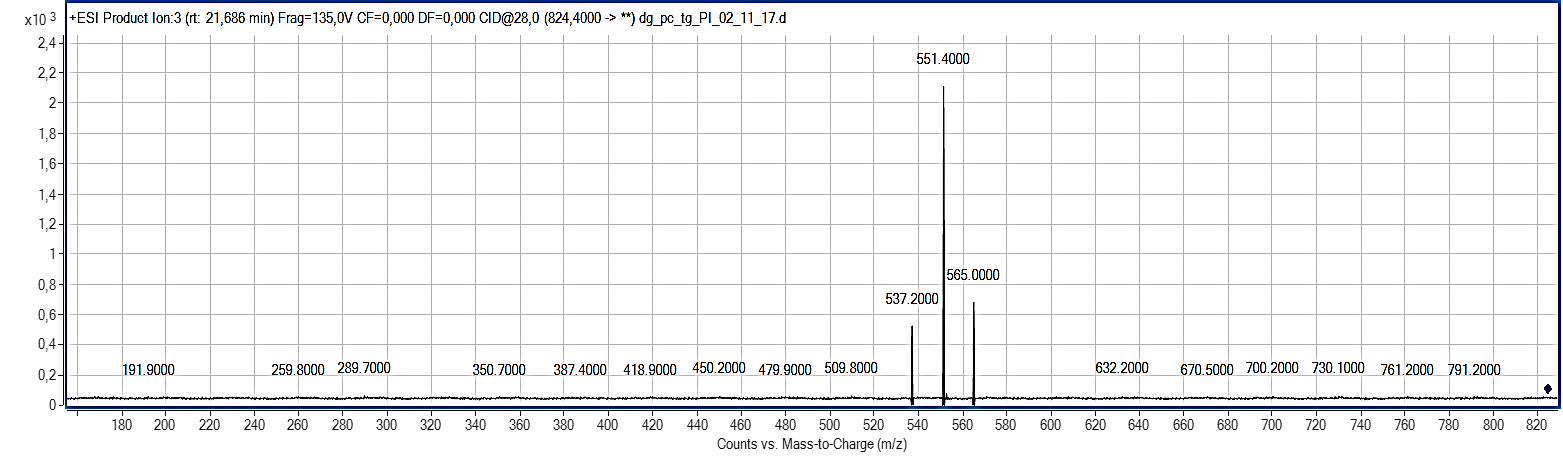


**Supplementary Figure 3 A-E**. EIC of [M+H]^+^ of TAG 54:3 (A) of pooled XfCFBP8402 cell extracts separated by RR-HPLC and detected by +ESI/TOF-MS. PI scan spectra of the standard (2 µM) TAG 54:3 (tri-oleoyl glycerol) (B) by LC-MS/MS (QQQ); PI scan spectra of TAG 54:3 (C), TAG 52:2 (D) and TAG 48:0 (E) in pooled XfCFBP8402 cell extracts by LC-MS/MS (QQQ). PI scan spectra of standard and bacterial TAG were obtained with the collision energy and fragmentor voltage set at 30 and 140 V, respectively.

A)

B)


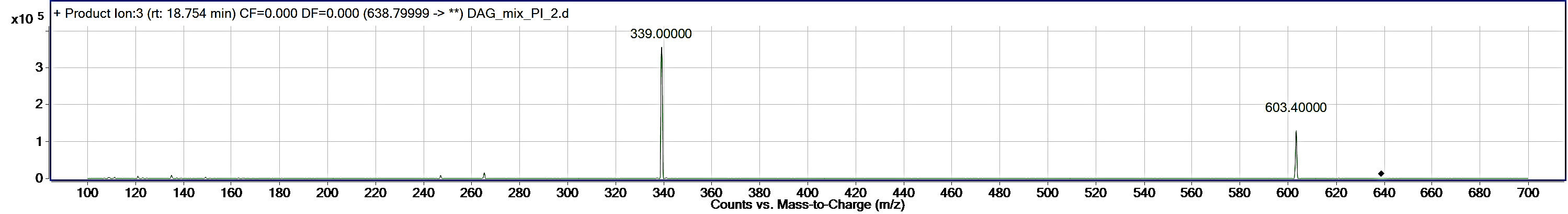
C)


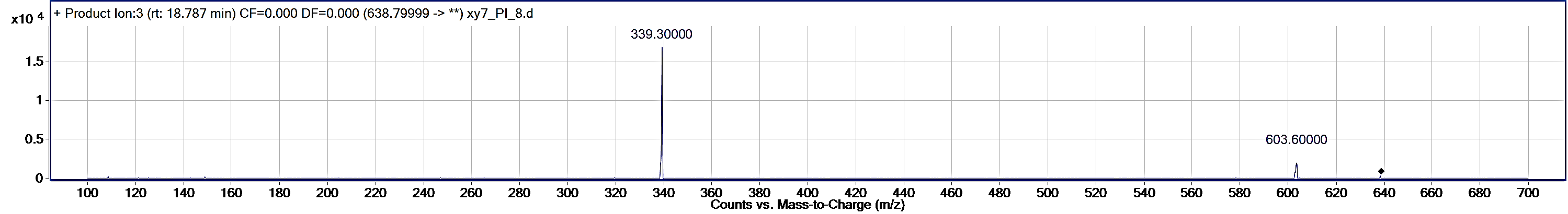
D)


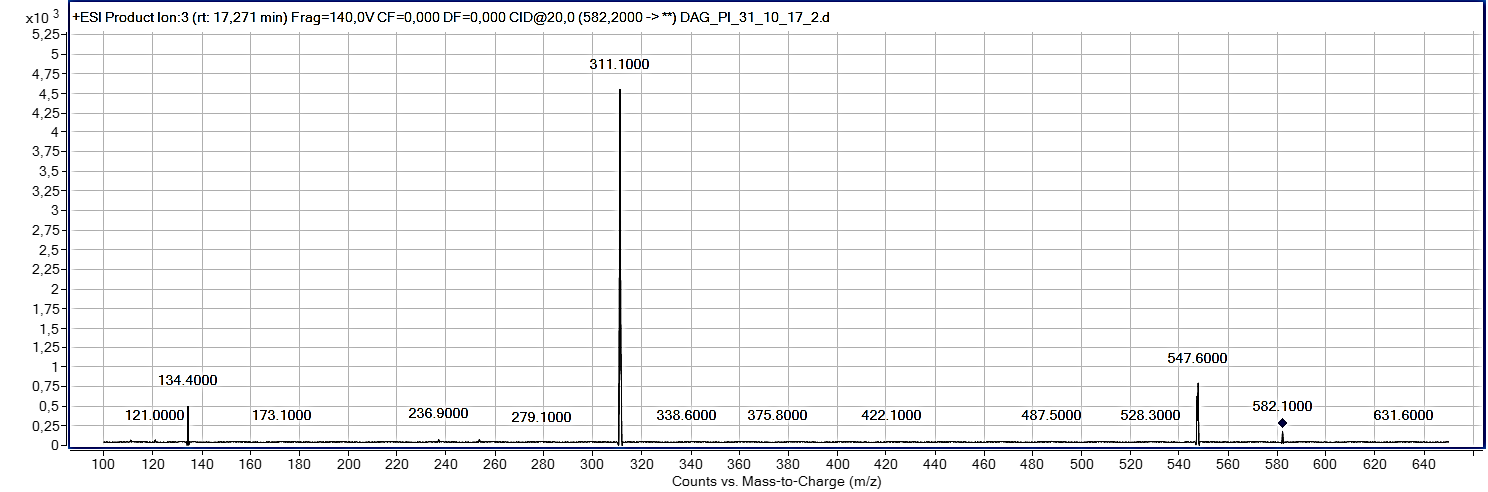


E)


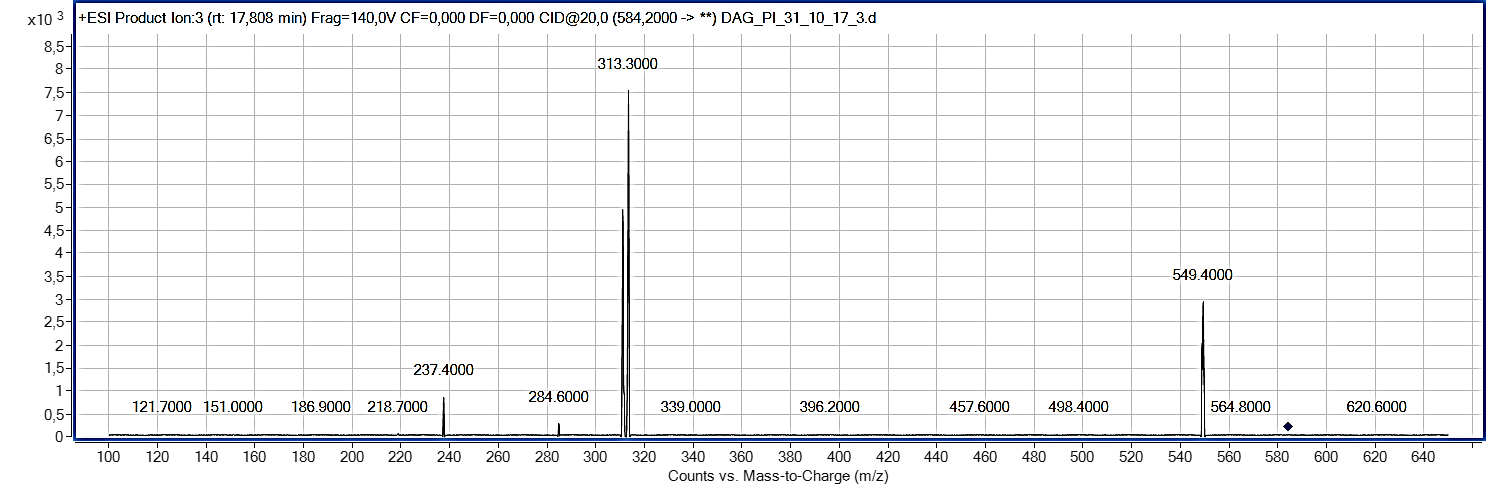


F)


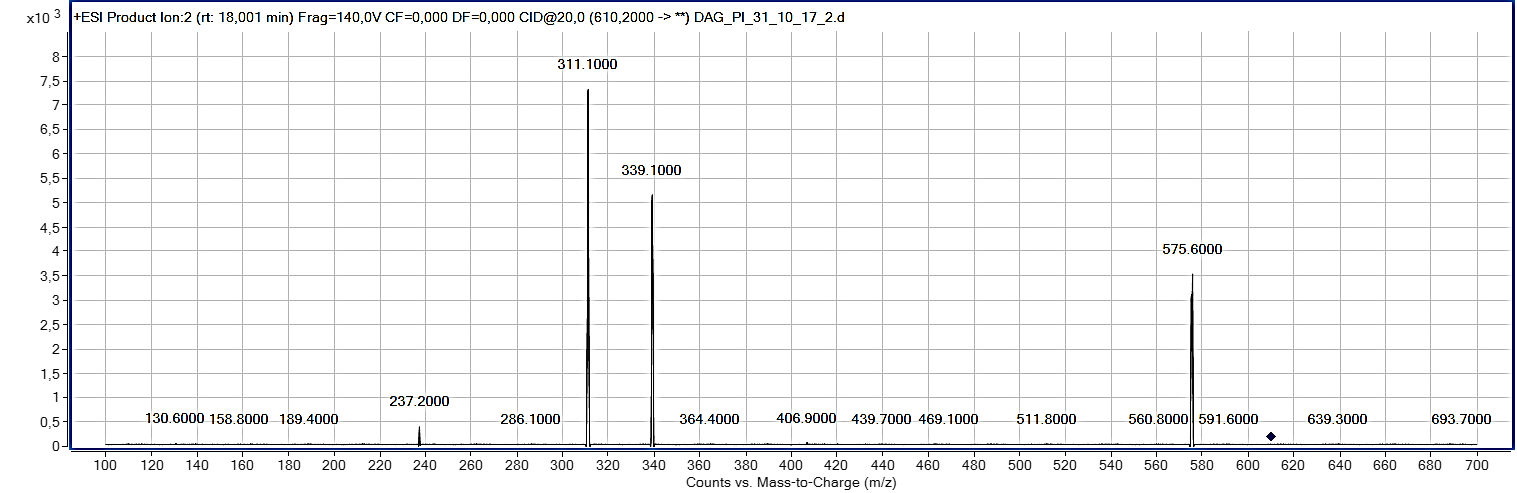


G)


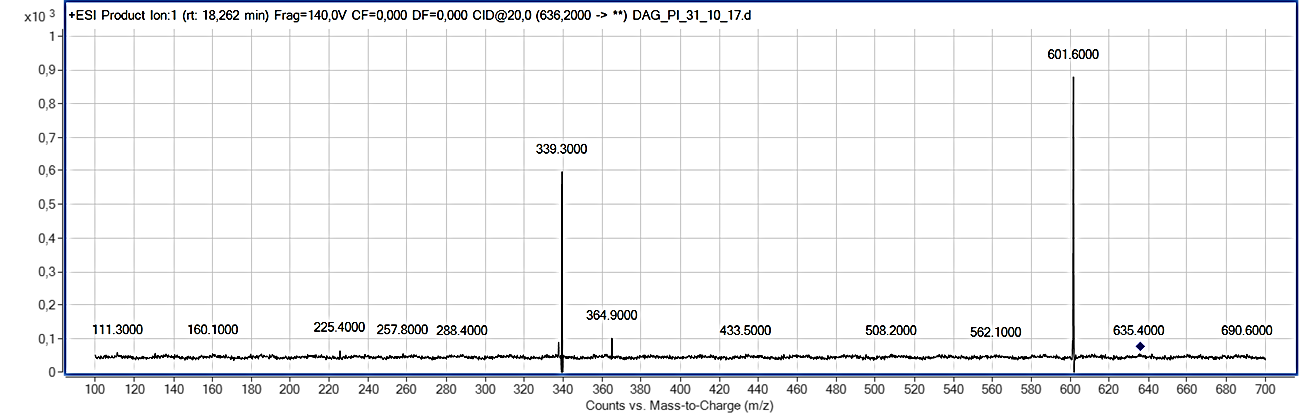


H)


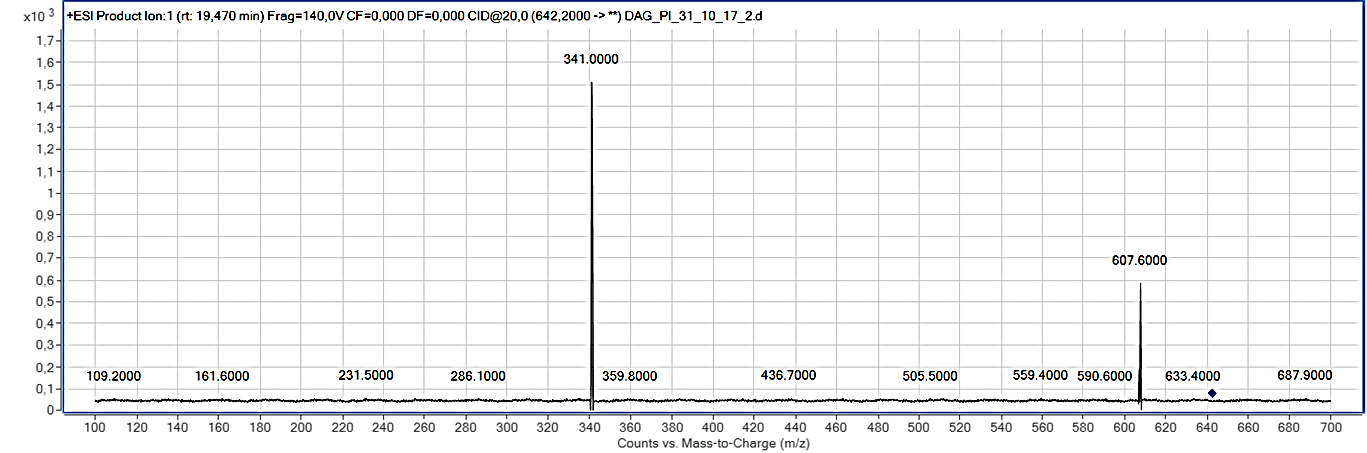


**Supplementary Figure 4 A-H**. EIC of [M+NH_4_]+ of DAG 36:2 (A) of pooled XfCFBP8402 cell extracts separated by RR-HPLC and detected by +ESI/TOF-MS. PI scan spectra of the standard (2 µM) DAG 36:2 (2-DIOLEYL-glycerol) by LC-MS/MS (QQQ); PI scan spectra of DAG 36:2 (C), DAG 32:2 (D), DAG 32:1 (E), DAG 34:2 (F), DAG 36:3 (G) and DAG 36:0 (H) in pooled XfCFBP8402 cell extracts by LC-MS/MS (QQQ). PI scan spectra of standard and bacterial DAG were obtained with the collision energy and fragmentor voltage set at 30 and 100 V, respectively.

A)


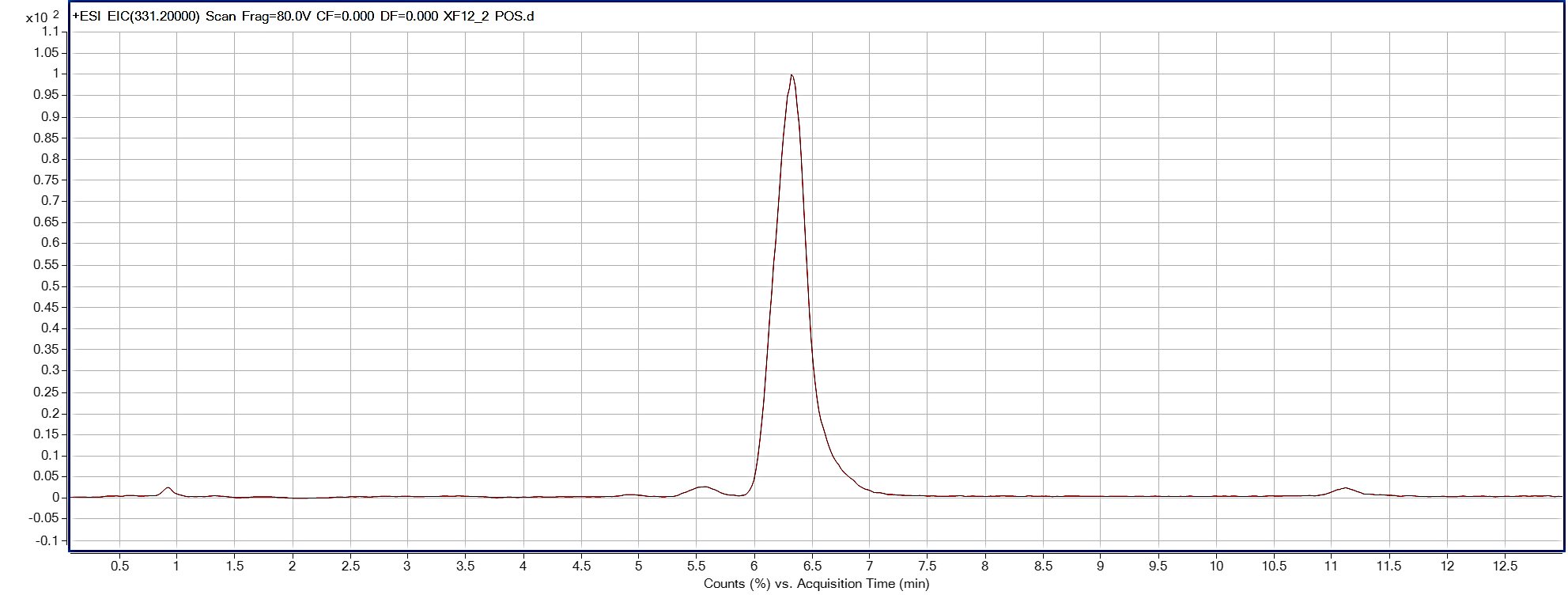


B)


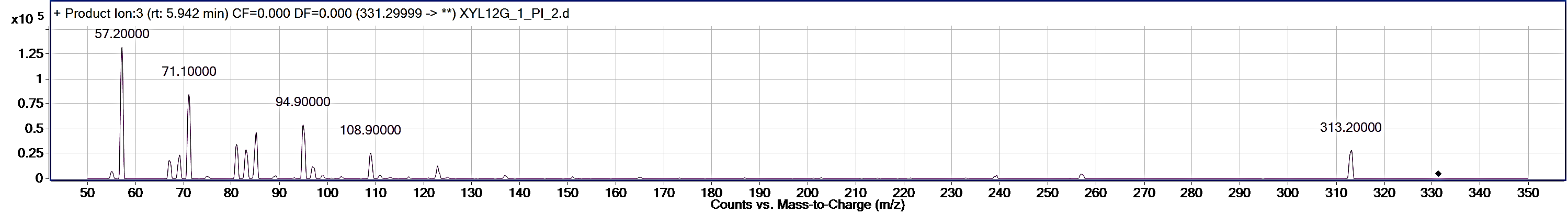


C)


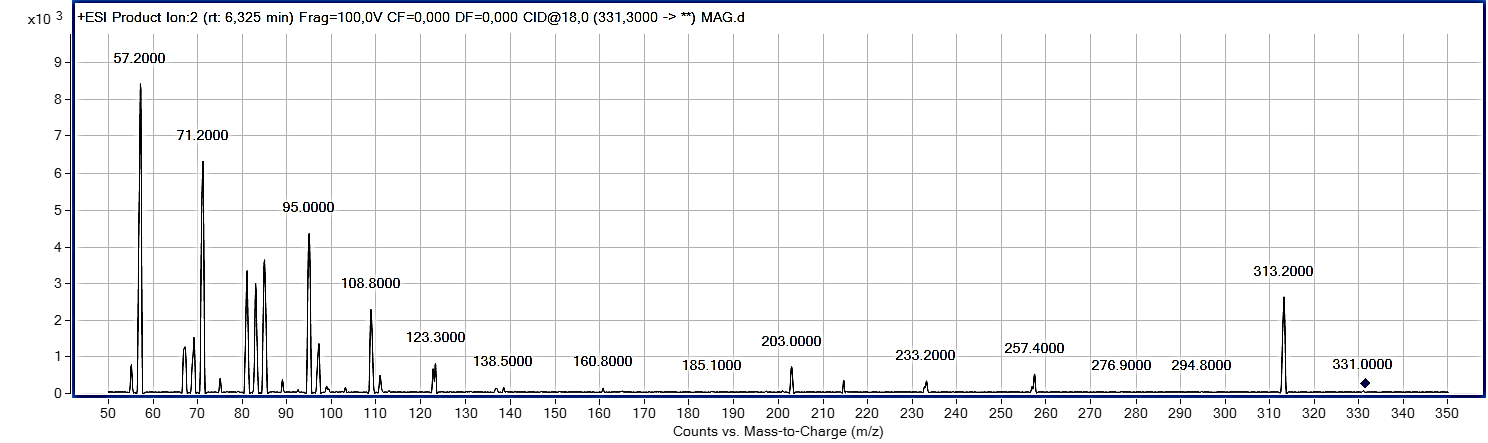


D)


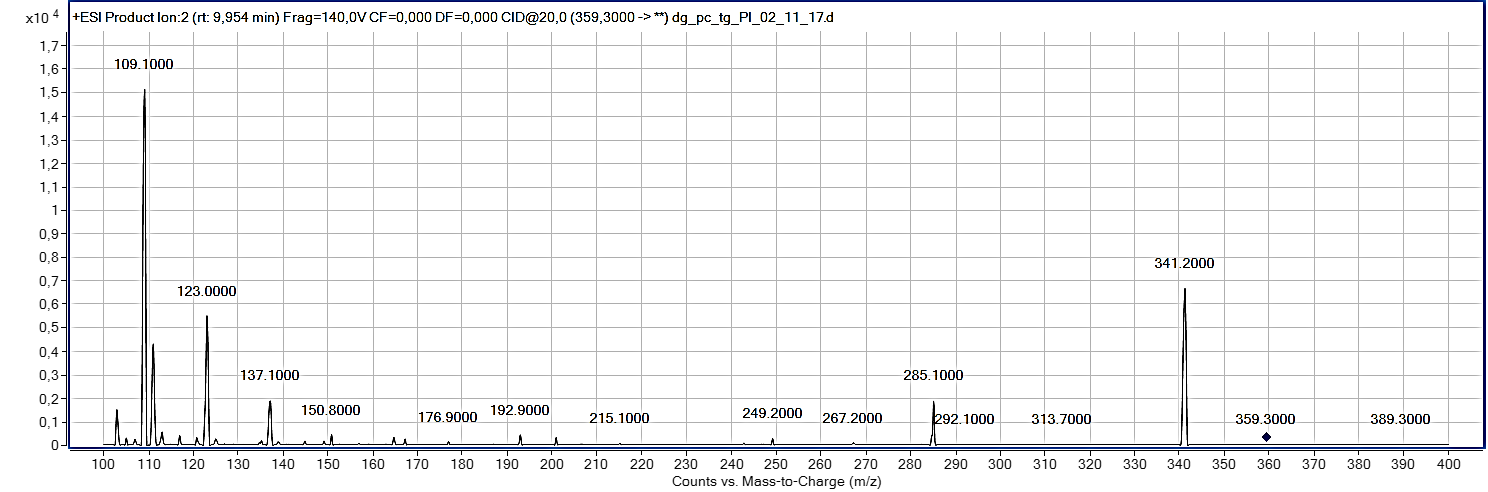


E)


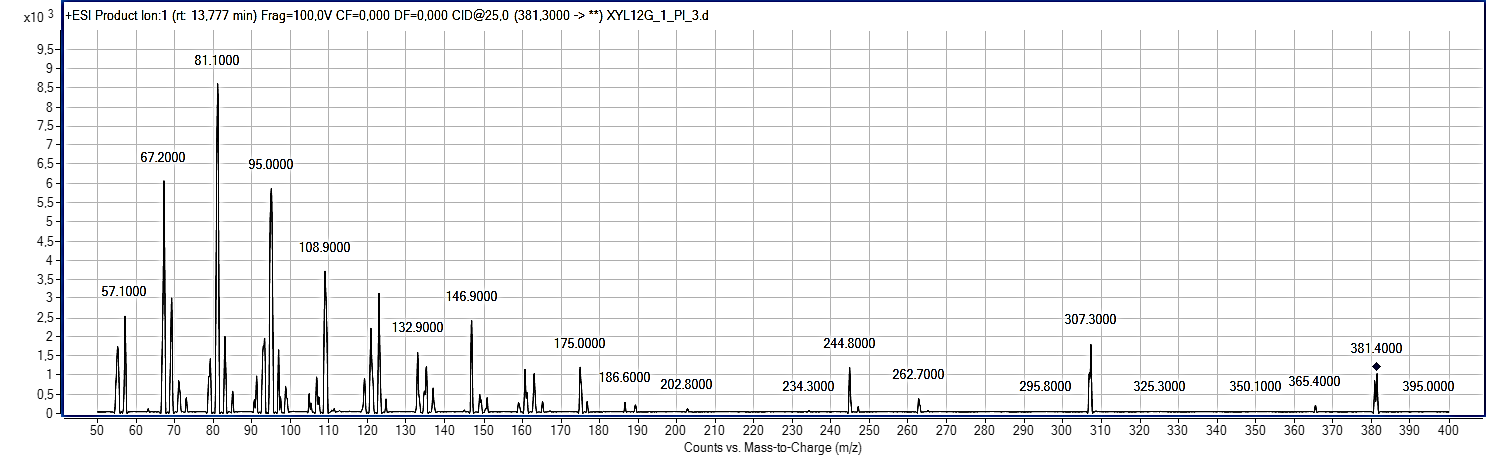


**Supplementary Figure 5 A-E**. EIC of [M+H]+ of MAG 16:0 (A) of pooled XfCFBP8402 cell extracts separated by RR-HPLC and detected by +ESI/TOF-MS. B) PI scan spectra of the standard (2 µM) MAG 16:0 (2-palmitoyl-glycerol) and LC-MS/MS (QQQ); (C) PI scan spectra of MAG 16:0, (D) MAG 18:0 and (E) MAG 20:3 in pooled XfCFBP8402 cell extracts by LC-MS/MS (QQQ). PI scan spectra of standard and bacterial MAG were obtained with the collision energy and fragmentor voltage set at 18 and 100 V, respectively

A)
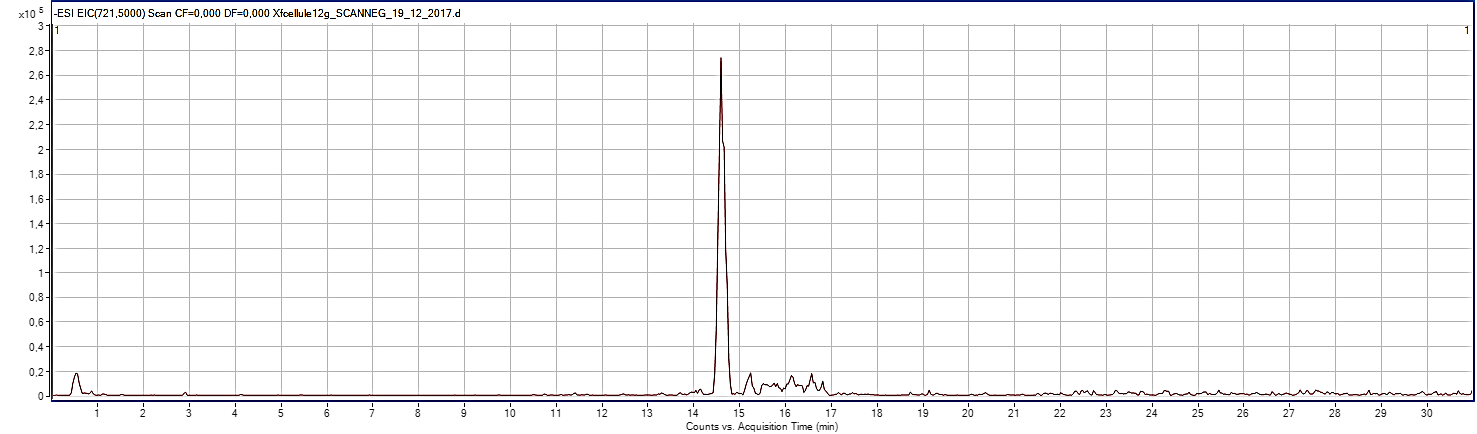


B)

C)

D)


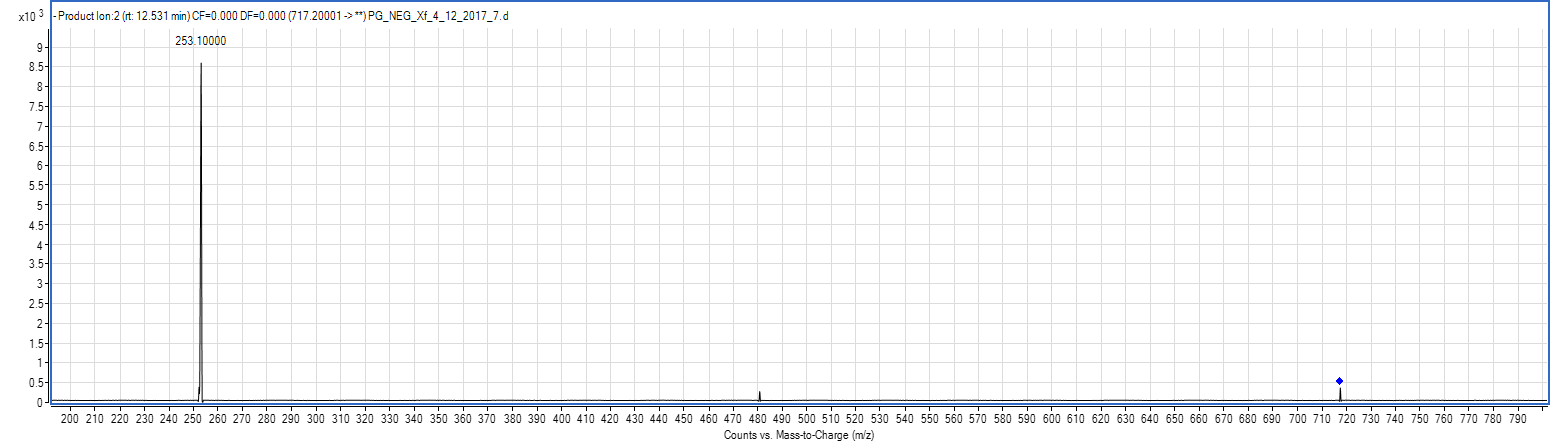


E)
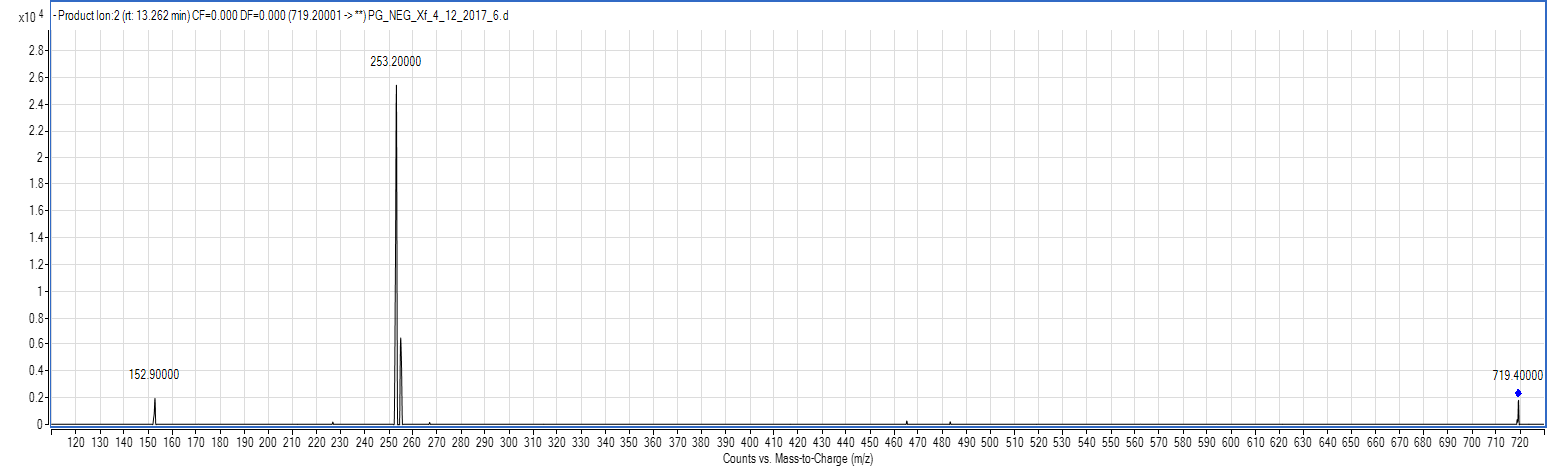


F)


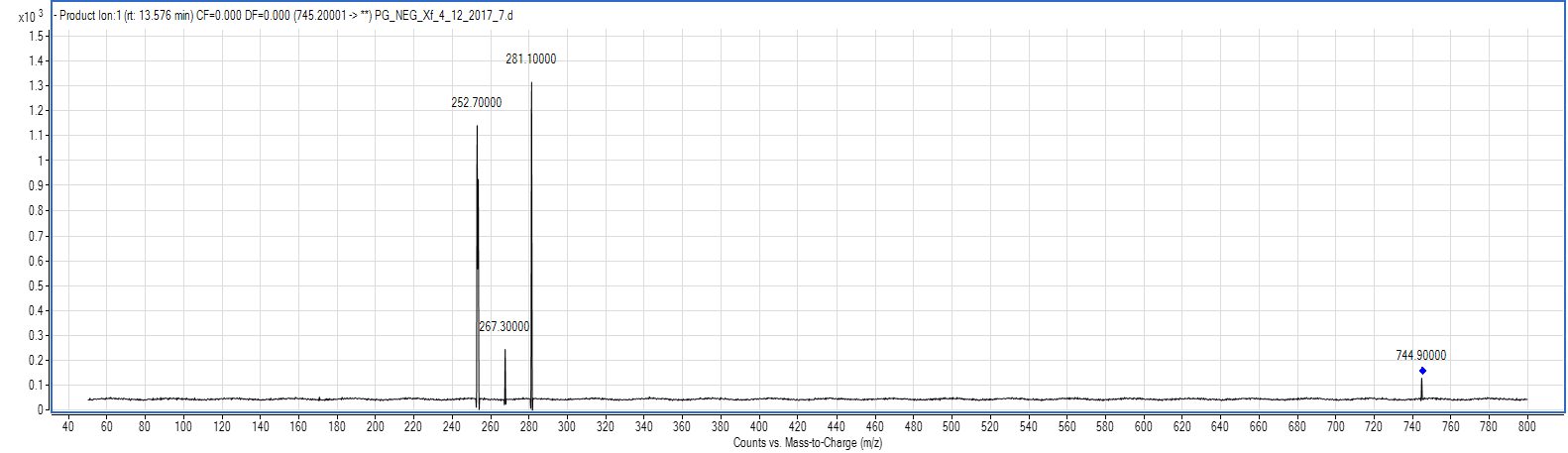


G)


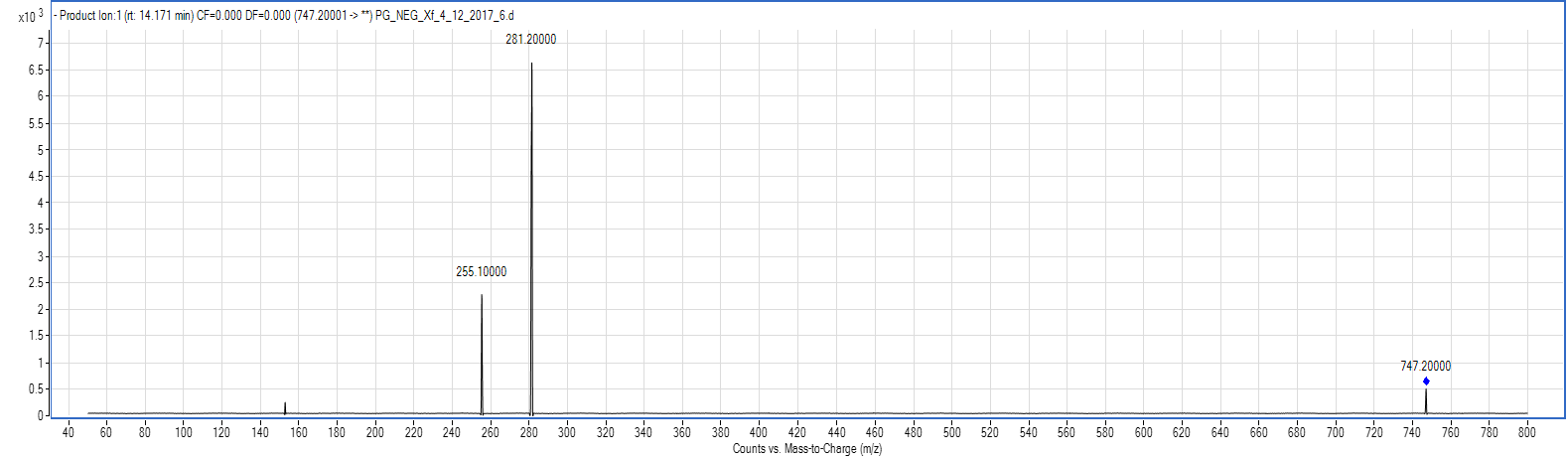

**Supplementary Figure 6 A-G.** EIC of [M-H]- of PG 16:0/16:0 (A) of pooled XfCFBP8402 cell extracts separated by RR-HPLC and detected by +ESI/TOF-MS. PI scan spectra of the standard (2 µM) PG 16:0/16:0 (**1,2-Dipalmitoyl-*sn*-glycero-3-phospho-*rac*-(1-glycerol**) (B) by LC-MS/MS (QQQ); PI scan spectra of PG 16:0/16:0 (C), PG 34:2 (D), PG 34:1 (E), PG 32:2 (F) and PG 32:1 (G) in pooled *XfCFBP8402* cell extracts by LC-MS/MS (QQQ). PI scan spectra of standard and bacterial PC were obtained with the collision energy and fragmentor voltage set at 30 and 135 V, respectively.

A)

B)


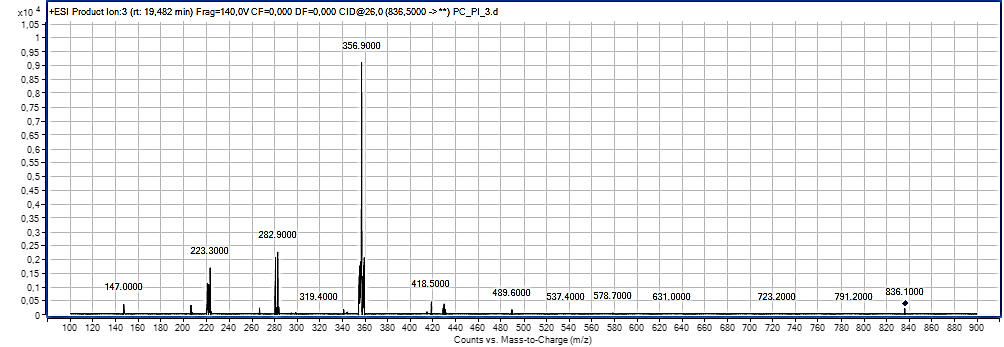


C)


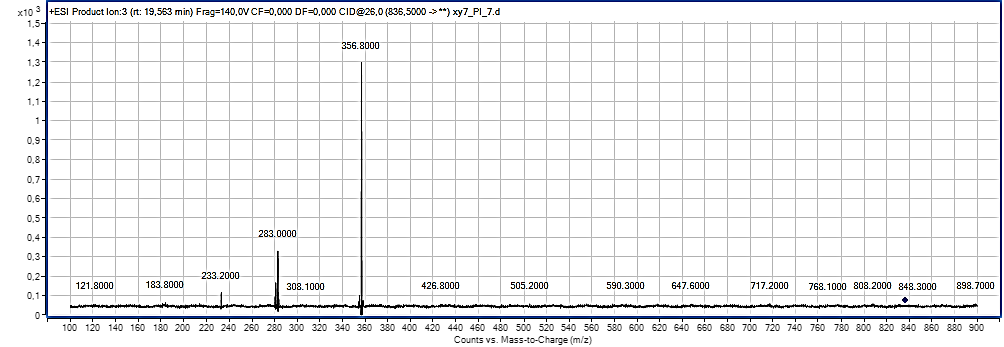


D)


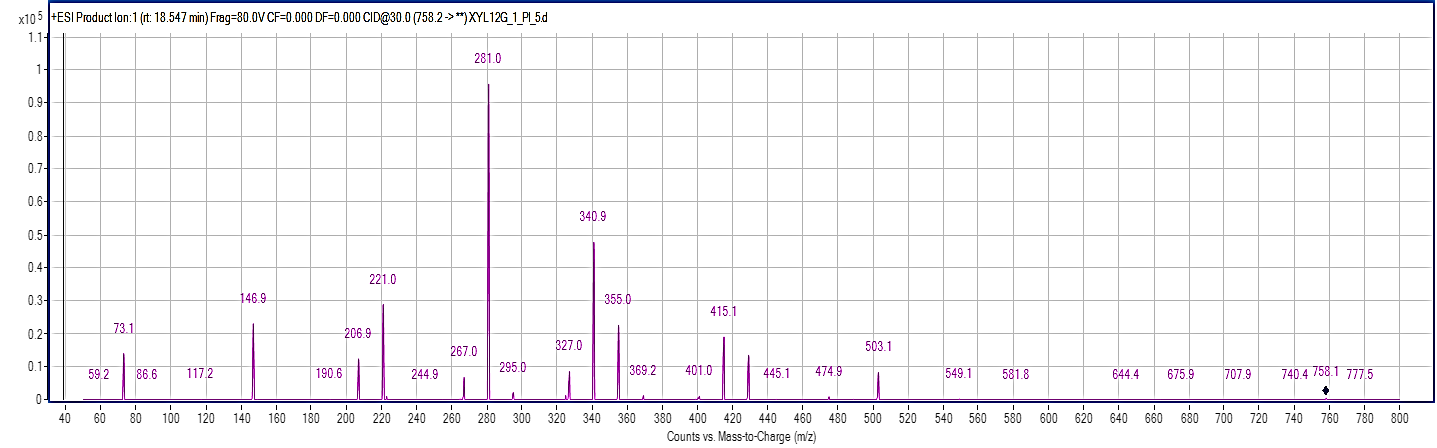


E)


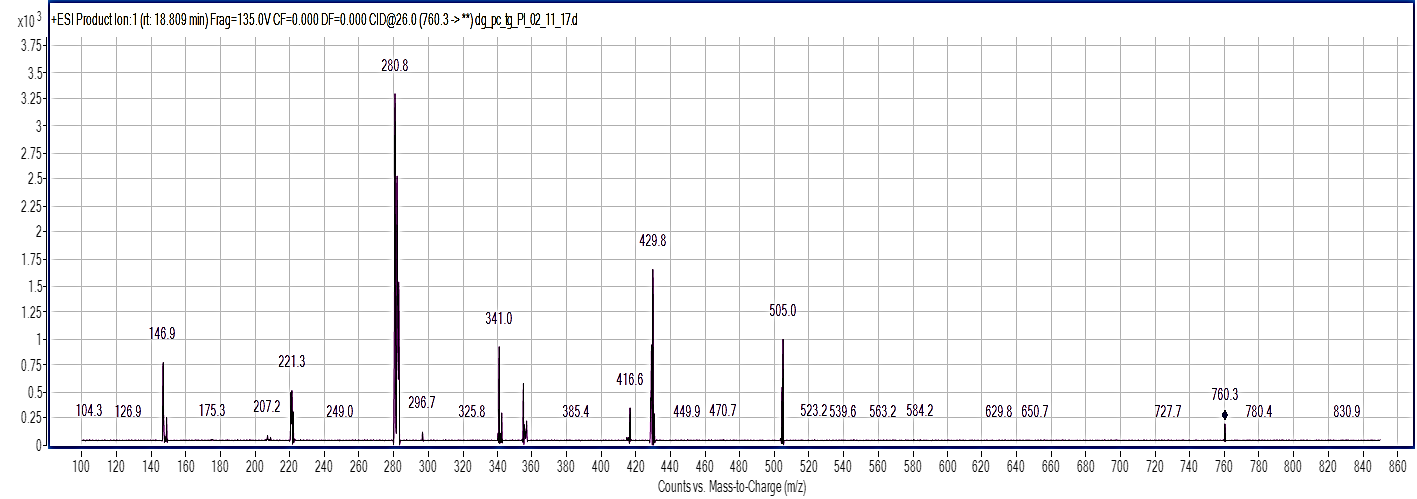


**Supplementary Figure 7 A-E**. EIC of [M+NH4]+ of PC 19:0/19:0 (A) of pooled XfCFBP8402 cell extracts separated by RR-HPLC and detected by +ESI/TOF-MS. PI scan spectra of the standard (2 µM) PC 19:0/19:0 (**1**,2-dinonadecanoyl-sn-glycero-3-phosphocoline) (B) by LC-MS/MS (QQQ); PI scan spectra of PC 38:0 (C), PC 34:2 (D) and PC 34:1 (E) in the pooled XfCFBP8402 cell extracts by LC-MS/MS (QQQ). PI scan spectra of standard and bacterial PC were obtained with the collision energy and fragmentor voltage set at 26 and 140 V, respectively

A)


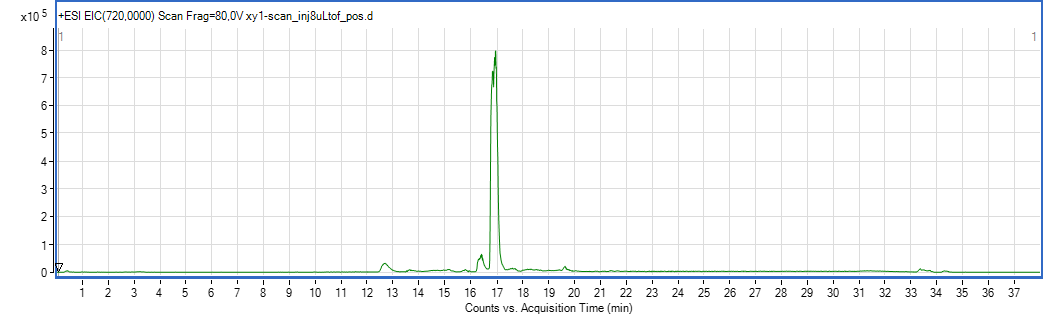


B)


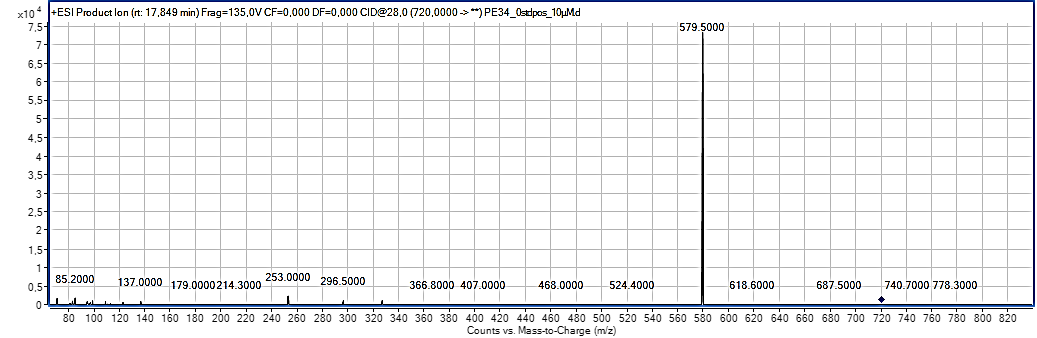


C)


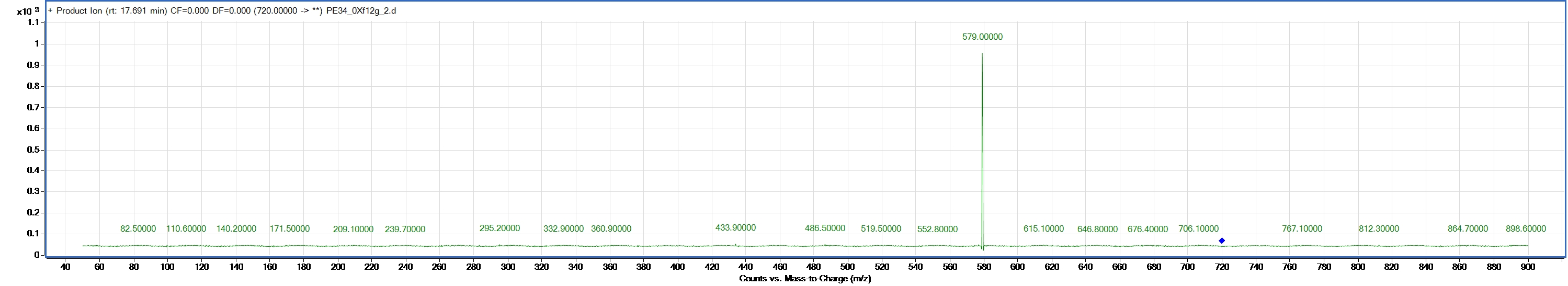


D)


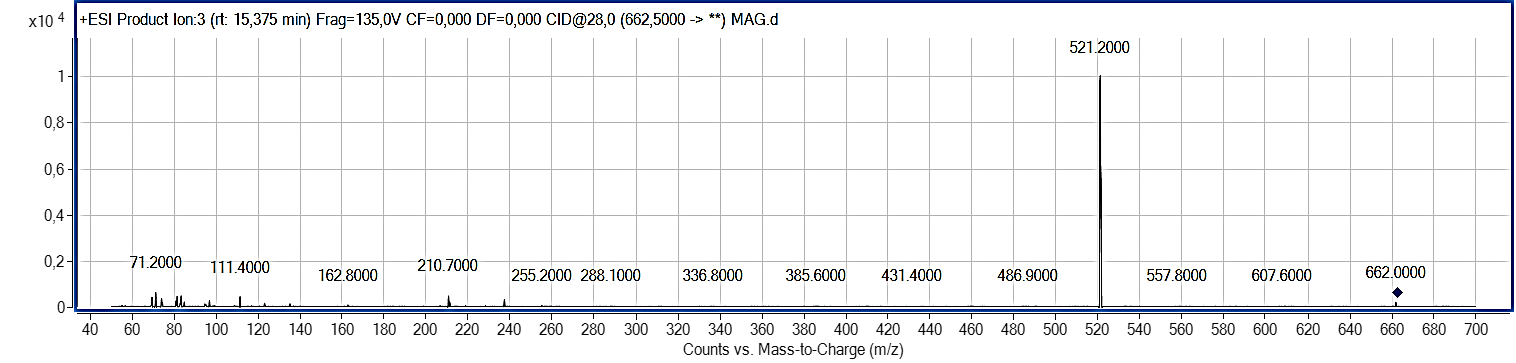


E)


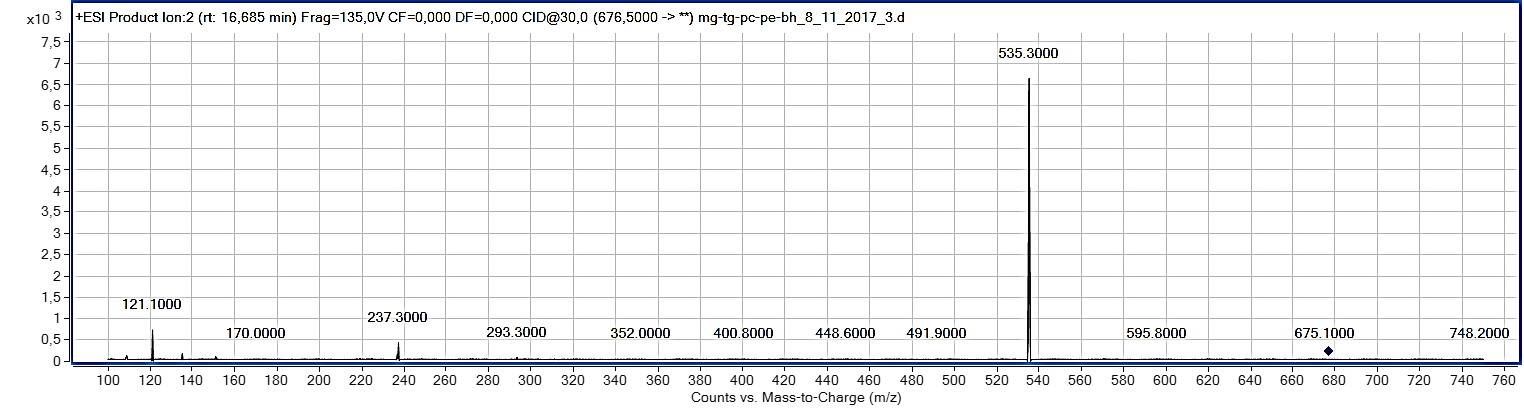


F)


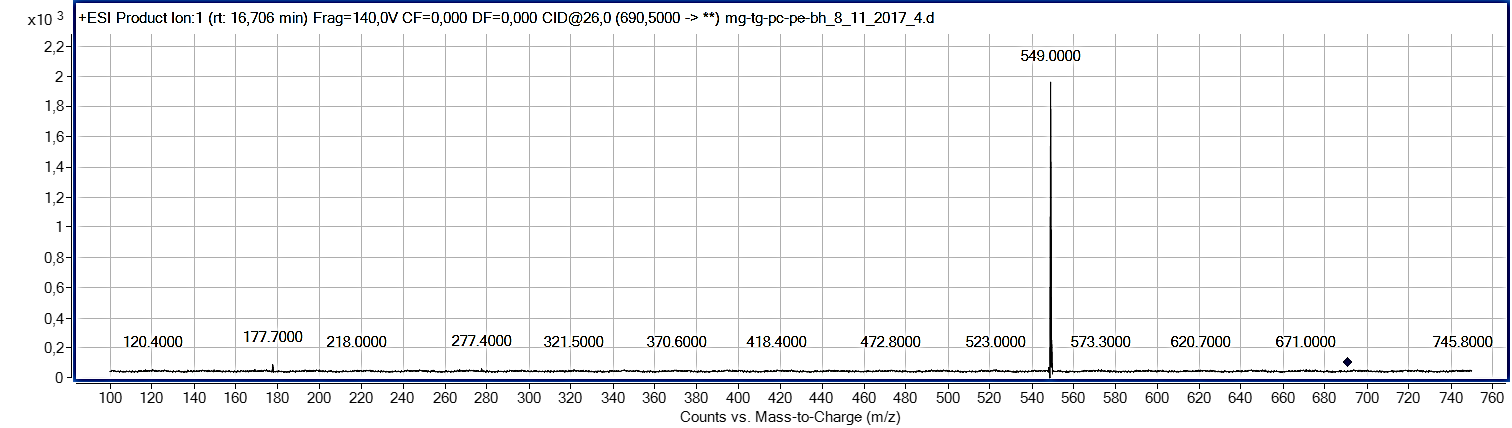


G)


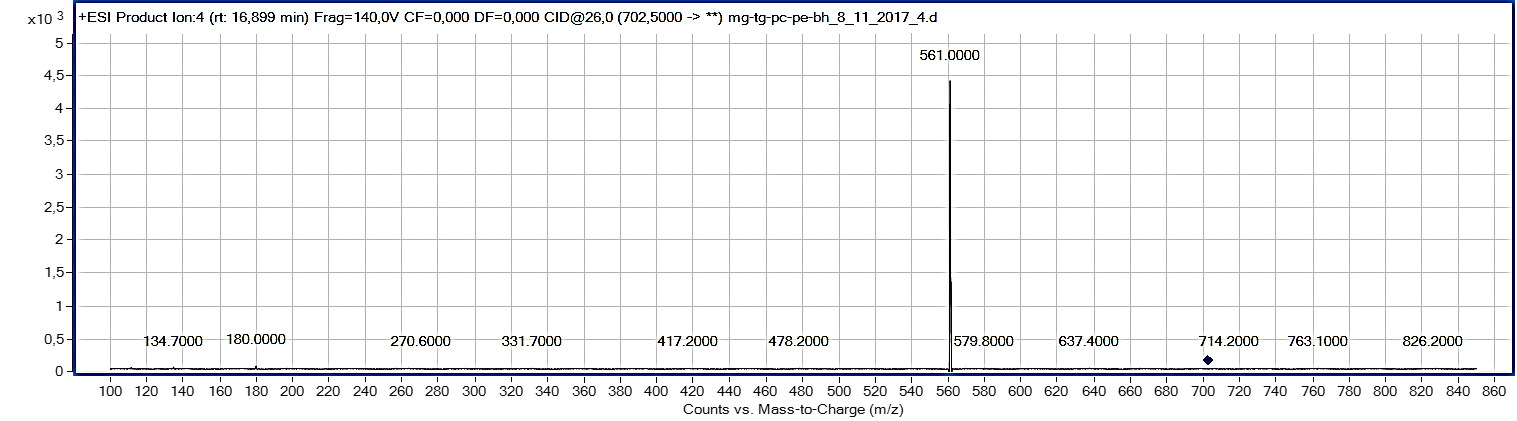


H)


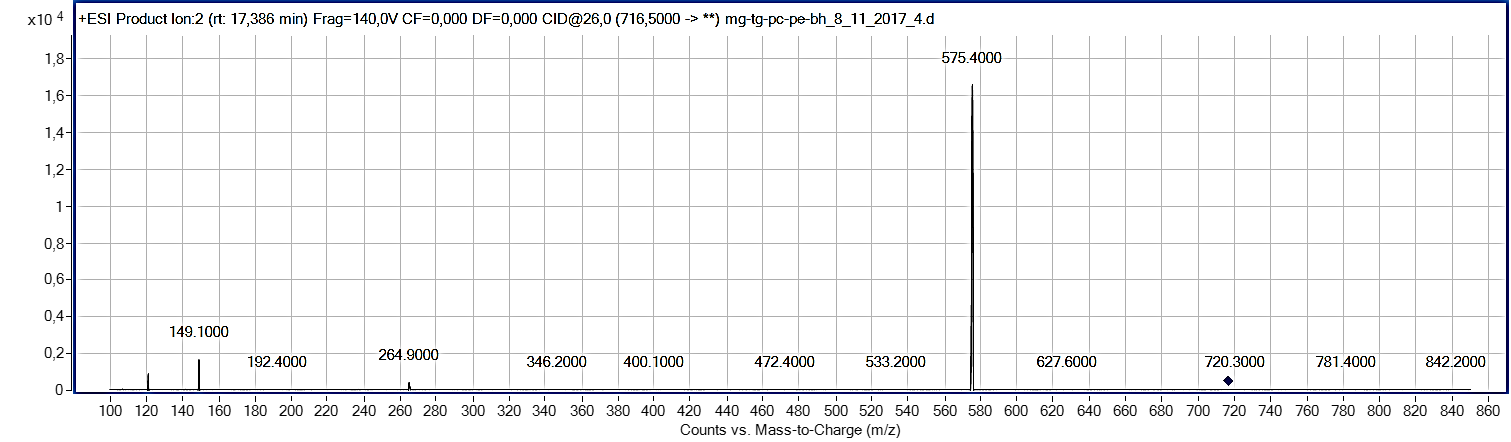


I)


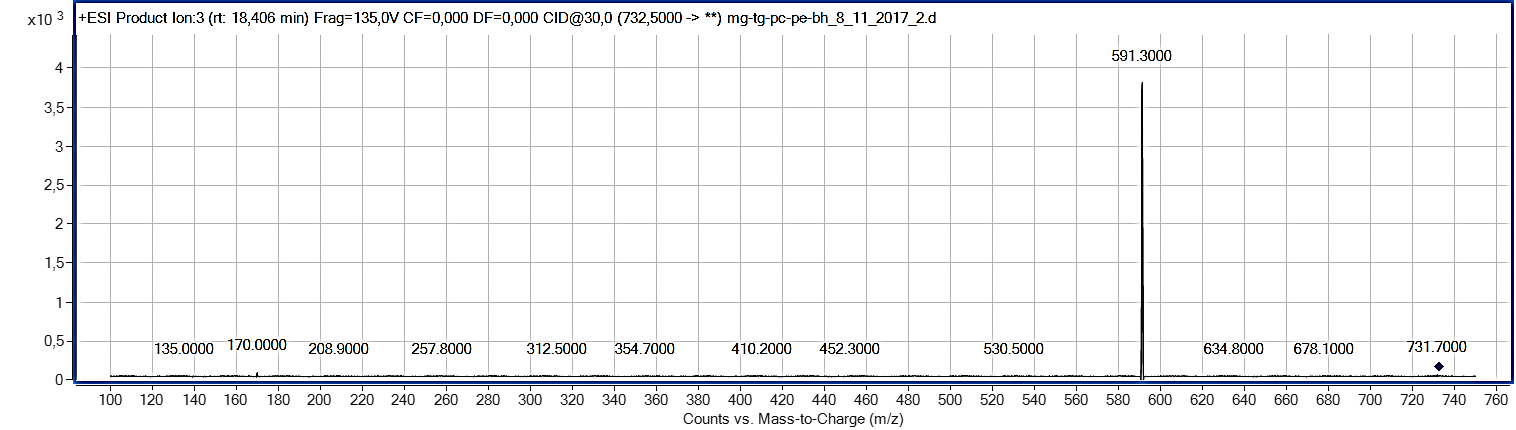


**Supplementary Figure 8 A-I**. EIC of [M-H]^-^ of PE 17:0/17:0 (A) of pooled XfCFBP8402 cell extracts separated by RR-HPLC and detected by +ESI/TOF-MS. PI scan spectra of the standard (10 µM) PE 17:0/17:0 (1,2-diheptadecanoyl-sn-glycero-3-phosphoethanolamine) (B) by LC-MS/MS (QQQ); PI scan spectra of PE 34:0 (C), PE O31:1/P31:0 (D), PE O32:1/P32:0 (E), PE O33:1/P33:0 (F), PE O34:2/P34:1 (G), PE 34:2 (H) and PE 35:1 (I) in pooled *XfCFBP8402* cell extracts by LC-MS/MS (QQQ). PI scan spectra of standard and bacterial PE were obtained with the collision energy and fragmentor voltage set at 28 and 135 V, respectively.

A)


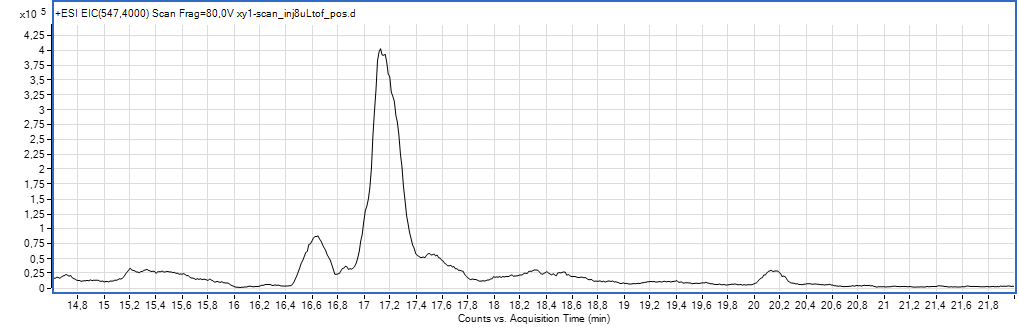


B)


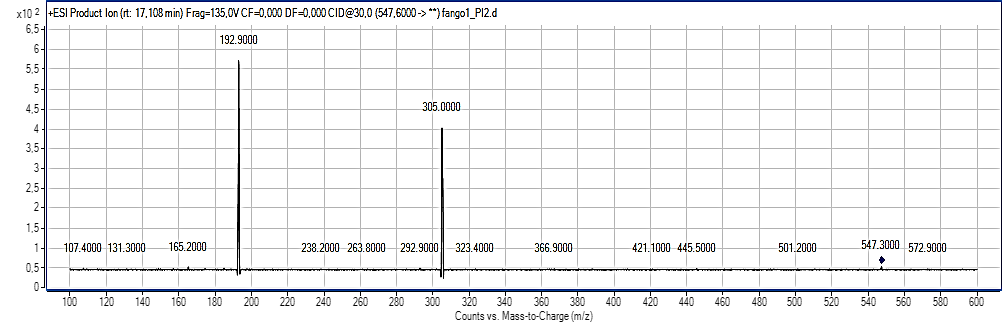


C)


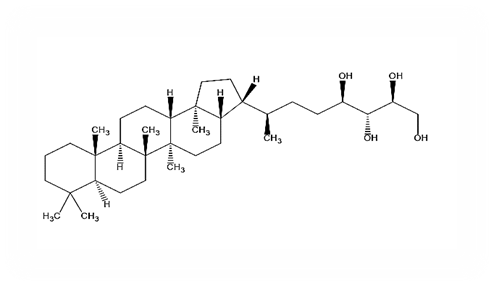

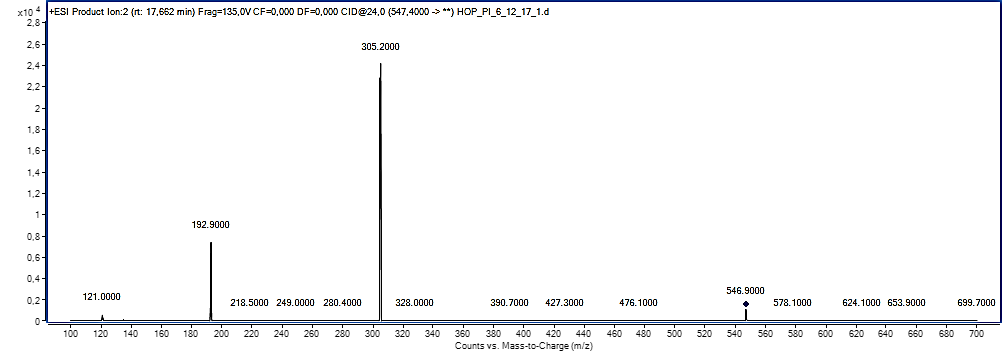


**Supplementary Figure 9A-C.** EIC of [M+H]^+^ of BHT (A) of pooled *XfCFBP8402* cell extracts separated by RR-HPLC and detected by +ESI/TOF-MS. PI scan spectra of the standard (2 µM) BHT (B) by LC-MS/MS (QQQ); PI scan spectra of BHT (C) in pooled *XfCFBP8402* cell extracts by LC-MS/MS (QQQ). PI scan spectra of standard and bacterial BHT were obtained with the collision energy and fragmentor voltage set at 25 and 80 V, respectively. In the box within the ESI product spectrum is presented the putative structure of BHT.

A)


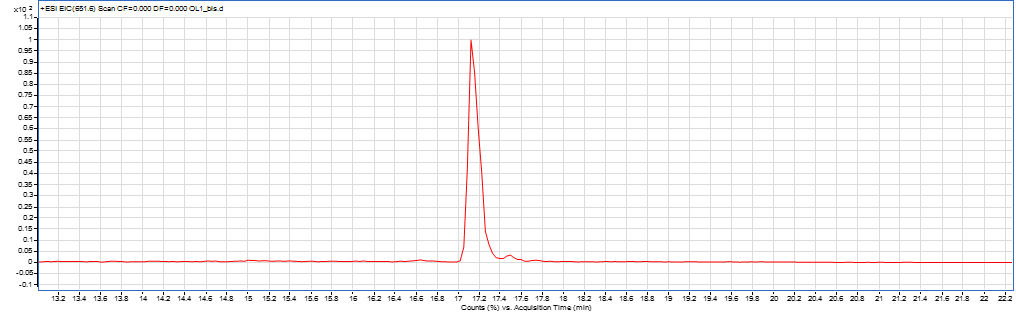


B)


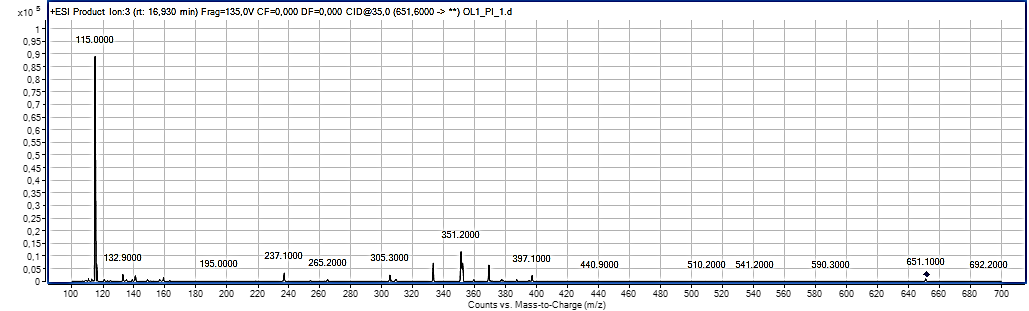


C)


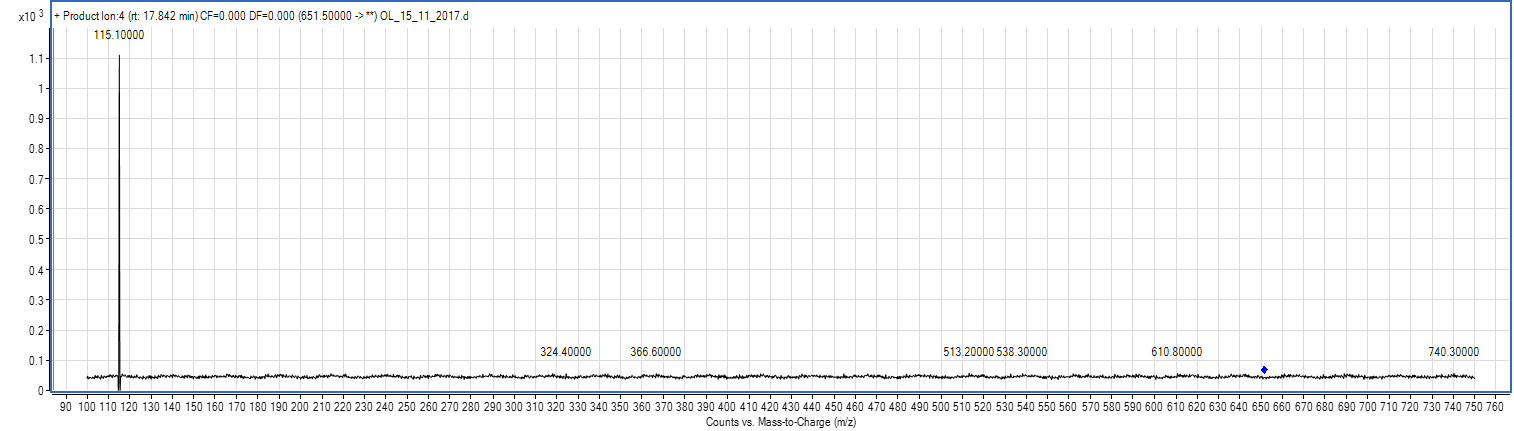


D)


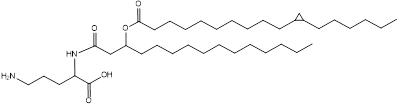

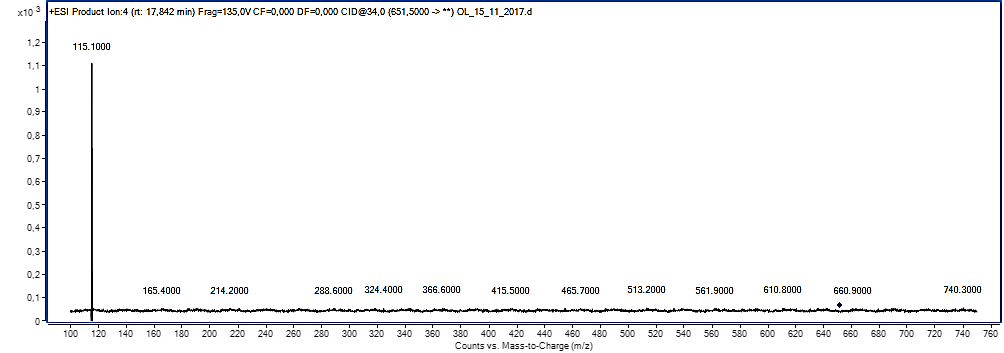


**Supplementary Figure 10 A-D**. EIC of [M+H]^+^ of OL1 (A) of pooled XfCFBP8402 cell extracts separated by RR-HPLC and detected by +ESI/TOF-MS. PI scan spectra of the standard (2 µM) OL1 (OL 15:0-OH/19:0 cyclo) by LC-MS/MS (QQQ) (B); PI scan spectra of OL1 (C) and OL (17:0/19:0) (D) in pooled XfCFBP8402 cell extracts by LC-MS/MS (QQQ). PI scan spectra of standard and bacterial PC were achieved with the collision energy and fragmentor voltage set at 35 and 135 V, respectively. In the box within the ESI product spectrum is presented the putative structure of BHT

**
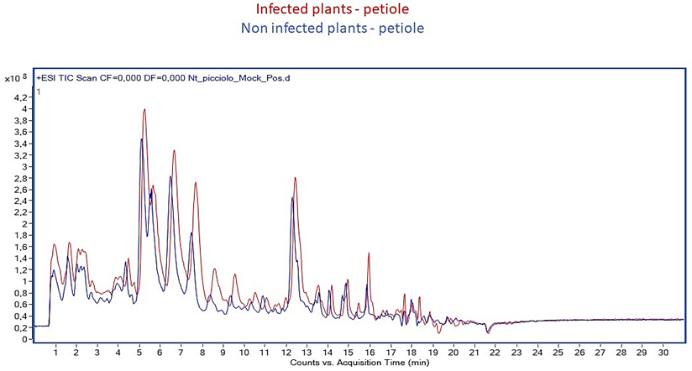

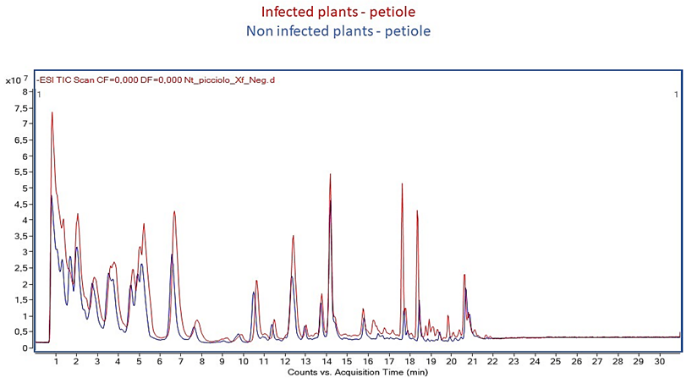

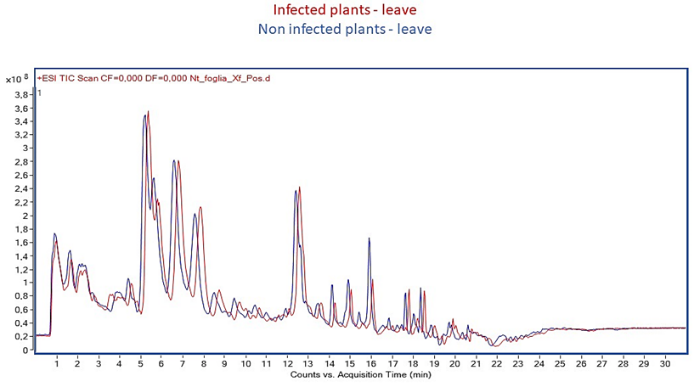

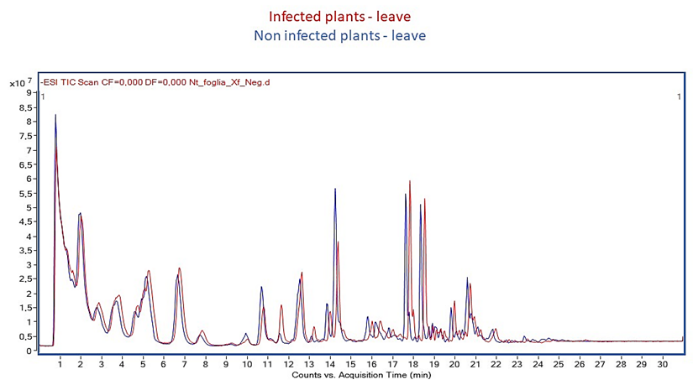

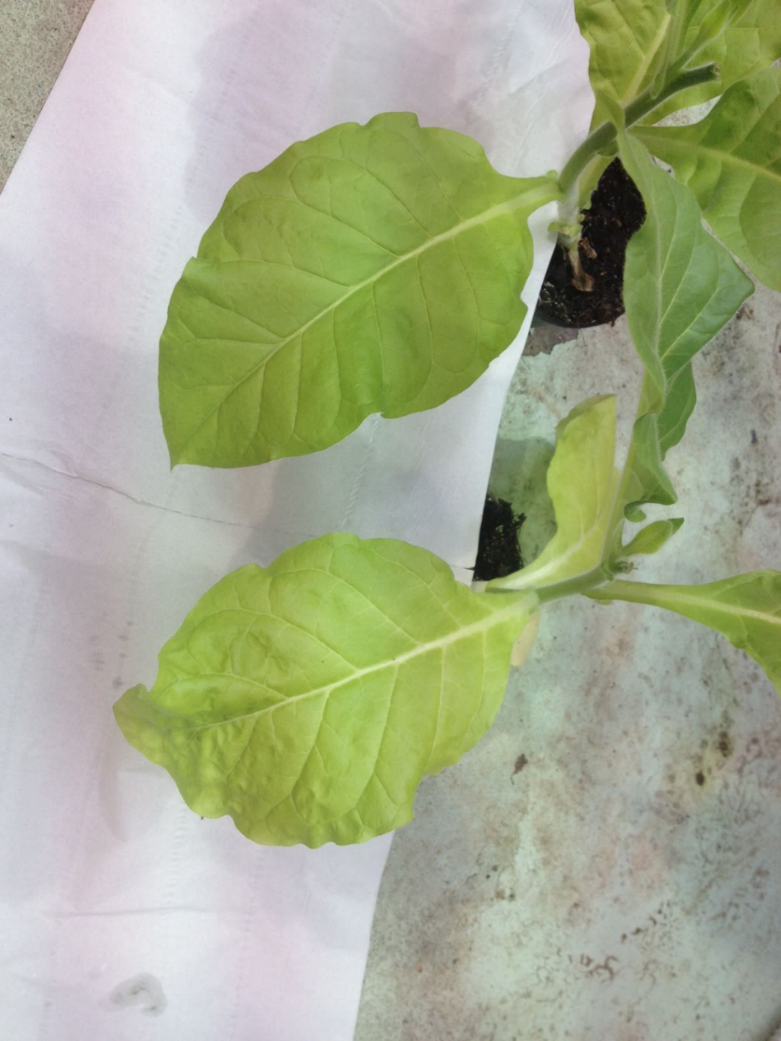
**

B

A

Mock

Xf-infected

**Supplementary Figure 11A, B.** A) Example of *Nicotiana tabacum* Petite Havana SR1 infected (Xf-infected) and non-infected (mock) with XfCFBP8402 at 15 days after inoculation. B) TIC chromatogram of TOF mass spectra acquired in positive and negative ion mode of *Nicotiana tabacum* Petite Havana SR1, leaves and petioles, infected (Xf-infected) and non-infected (mock) with XfCFBP8402 at 30 days after inoculation

**Supplementary TABLE 1A.** Summary of precursor ion scanning for sample acquisition in negative and positive ion mode for PE, PC and OL.

| entity group name | ion search | precursor ion/neutral loss* | MS1 from | polarity | collision energy | fragmentor voltage | Scan time (ms) |
| --- | --- | --- | --- | --- | --- | --- | --- |
| ornithine lipids | [M-H]^-^ | 131.2 | 300🡪1000 | negative | 40 | 135 | 500 |
| ornithine lipids | [M+H-H_2_O]^+^ | 115.2 | 300🡪1000 | positive | 15 | 75 | 500 |
| PE | [M+H]^+^ | 141.1* | 300🡪1000 | positive | 36 | 140 | 500 |
| PC | [M+H]^+^ | 184.2 | 300🡪1000 | positive | 36 | 140 | 500 |

Scan time unit= milliseconds (ms); *searched by neutral loss

**Supplementary TABLE 1B.** Summary of fragment ion scanning for characterizing mass fragmentation pattern of standard lipid compounds for lipid compounds representative of BHP, OL, MAG, DAG, TAG, PC, PE, PG.

| entity name | product ion (m/z) | MS2 from | polarity | collision energy | fragmentor voltage | Scan time (ms) | retention time |
| --- | --- | --- | --- | --- | --- | --- | --- |
| BHT | 547.5 | 50🡪600 | positive | 26 | 135 | 500 | 15.4 |
| OL3-OH (18:1/19:1) | 691.2 | 50🡪700 | positive | 40 | 135 | 500 | 16.4 |
| MAG (16:0) | 330.5 | 50🡪350 | positive | 16 | 100 | 500 | 6.3 |
| MAG (18:0) | 358.6 | 50🡪400 | positive | 18 | 100 | 500 | 6.7 |
| DAG 36:2 (18:1/18:1) | 620.9 | 50🡪650 | positive | 30 | 100 | 500 | 10.3 |
| TAG 54:3 (18:1/18:1/18:1) | 885.4 | 50🡪950 | positive | 30 | 140 | 500 | 22.2 |
| PC (19:0/19:0) | 836.5 | 50🡪850 | positive | 26 | 140 | 500 | 19.6 |
| PE (17:0/17:0) | 720.0 | 50🡪750 | positive | 26 | 135 | 500 | 17.6 |
| PG (16:0/16:0) | 721.9 | 50🡪800 | negative | 28 | 140 | 500 | 15.0 |

**Supplementary TABLE 1C**. List of FFA analyzed in SIM mode with their respective m/z and retention times.

| FA | Mass  [M-H]^-^ | RT (min) |
| --- | --- | --- |
| C24:0 | 367.3 | 16.5 |
| C24:1 | 365.3 | 15.5 |
| C22:0 | 339.3 | 15.2 |
| C22:1 | 337.3 | 13.8 |
| C20:0 | 311.2 | 13.4 |
| C20:1 | 309.2 | 11.6 |
| C20:2 | 307.3 | 9.7 |
| C19:0 | 297.2 | 12.1 |
| C18:0 | 283.2 | 10.8 |
| C18:1 | 281.2 | 7.8 |
| C18:2 | 279.2 | 5.2 |
| C18:3 | 277.2 | 3.7 |
| C17:1 | 267.2 | 7.6 |
| C17:0 | 269.2 | 9.1 |
| C16:0 | 255.2 | 6.3 |
| C16:1 | 253.2 | 3.9 |
| C15:1 | 239.2 | 3.0 |
| C15:0 | 241.2 | 4.5 |
| C14:1 *cis* | 225.2 | 2.7 |
| C14:1 *trans* | 225.2 | 2.1 |
| C14:0 | 227.2 | 3.3 |
| C13:0 | 213.2 | 2.3 |
| C12:0 | 199.2 | 1.7 |

**Supplementary TABLE 3A.** List of lipid entities analyzed in MRM mode with their respective mass transition, retention time, collision energy and fragmentor voltage values.

| Compound  Name | RT (min) | MRM transition  (m/z) | Fragmentor Voltage | Collision Energy | Scan time (ms) |
| --- | --- | --- | --- | --- | --- |
| MAG 18:0 | 6.8 | 331.2 -> 313.2 | 100 | 18 | 200 |
| MAG 20:0 | 11.0 | 359.2 -> 341.2 | 100 | 25 | 200 |
| PG 32:1 | 14.6 | 719.2 -> 253.2 | -140 | 28 | 200 |
| PG 34:1 | 14.9 | 747.2 -> 281.2 | -140 | 28 | 200 |
| PE O34:2 (PE P34:1) | 16.8 | 690.5 -> 549.5 | 140 | 26 | 200 |
| PE 34:2 | 17.0 | 716.4 -> 575.4 | 140 | 26 | 200 |
| BHT | 17.5 | 547.5 -> 305.2 | 135 | 24 | 200 |
| DAG 32:2 | 17.7 | 582.2 -> 311.2 | 140 | 20 | 200 |
| DAG 34:2 | 17.7 | 610.2 -> 339.2 | 140 | 20 | 200 |
| TAG 48:0 | 18.2 | 824.4 -> 551.4 | 130 | 28 | 200 |
| OL 34:0 | 18.4 | 651.5 -> 115.1 | 135 | 28 | 200 |
| PC 34:2 | 19.2 | 758.2 -> 281.2 | 80 | 30 | 200 |
| TAG 52:2 | 21.8 | 876.4 -> 577.4 | 135 | 28 | 200 |

**Supplementary TABLE 3B.** List of oxylipins analyzed in MRM mode with their respective mass transition, retention time, collision energy and fragmentor voltage values.

| Compound  Name | RT (min) | MRM transition  (m/z) | Fragmentor Voltage | Collision Energy | Scan time (ms) |
| --- | --- | --- | --- | --- | --- |
| Jasmonic acid | 5.8 | 209.2→59.1 | 135 | 28 | 500 |
| methyl-jasmonate | 8.4 | 223.1→75.2 | 135 | 25 | 500 |
| 12,13 - diHOME | 12.4 | 313.3→183.1 | 140 | 18 | 500 |
| 9,10 - diHOME | 12.8 | 313.3→201.1 | 140 | 14 | 500 |
| 8,13 - diHODE | 13.0 | 311.3→171.2 | 140 | 14 | 500 |
| 9 – HOTrE | 14.3 | 293.2→171.2 | 140 | 18 | 500 |
| 13 - HOTrE | 14.3 | 293.2→195.2 | 140 | 18 | 500 |
| 10-HOME | 14.4 | 297.2→155.2 | 135 | 36 | 500 |
| 9-oxoOTrE | 14.7 | 291.4→185.2 | 140 | 18 | 500 |
| 8 – HODE | 15.3 | 295.2→157.2 | 140 | 20 | 500 |
| 13 – HODE | 15.7 | 295.2→195.2 | 140 | 20 | 500 |
| 13-HpOTrE | 15.7 | 309.2→223.2 | 60 | 6 | 500 |
| 10 – HODE | 15.7 | 295.2→183.2 | 140 | 20 | 500 |
| 9 – HODE | 15.8 | 295.4→171.2 | 140 | 20 | 500 |
| 9 - HODEd4 (**ISTD**) | 15.8 | 299.2→172.2 | 140 | 20 | 500 |
| 9-HpOTrE | 15.9 | 309.2→185.2 | 60 | 6 | 500 |
| 13 - HPODE | 16.2 | 311.1→113.2 | 80 | 14 | 500 |
| 8 – HPODE | 16.2 | 311.1→171.2 | 80 | 14 | 500 |
| 9 – oxoODE | 16.3 | 293.2→185.2 | 140 | 18 | 500 |
| 11 – HPODE | 16.3 | 311.2→197.2 | 80 | 14 | 500 |
| 9 – HPODE | 16.3 | 311.2→185.2 | 80 | 14 | 500 |
| 10-HpOME | 16.3 | 313.2→185.2 | 60 | 30 | 500 |
| 13 – oxoODE | 16.4 | 293.2→113.2 | 140 | 20 | 500 |
| 12(13)-EpOME | 17.8 | 295.2→195.2 | 140 | 14 | 500 |
| 9(10)-EpOME | 18.1 | 295.2→171.2 | 140 | 18 | 500 |

**Supplementary TABLE 3C.** Compound name and structures of the main oxylipins found in our samples (cell extract and culture filtrates of XfCFBP8402)

| Name | Structure |
| --- | --- |
| 10 HpOME |  |
| 7,10 diHOME |  |
| 9,10 di HOME |  |
| 10 HOME |  |
| 9,10 epOME |  |
| 13 HODE |  |
| 8,13 diHODE |  |
| 13 HOTre |  |

| **Supplementary Table 5**. Relative abundance ISTD-normalized (tricosanoic acid) of different FFA in bacterial cells, culture filtrate and plant tissues infected (XfCFBP8402 -infected) and non-infected (mock). | | | | | | | | |
| --- | --- | --- | --- | --- | --- | --- | --- | --- |
|  | FFA (relative abundance ISTD-normalized) | | | | | | | |
|  | 7 DAI_cells | 11 DAI_cells | 7 DAI _CF | 11 DAI _CF | *N. tabacum* (mock) | | *N. tabacum* (XfCFBP8402 -infected) | |
|  |  |  |  |  | *Petiole* | *Leaves* | *Petiole* | *Leaves* |
| 12:0 | 2059185.5 ± 31796.5 | 72537.0 ± 3850.8 | 159066.7±46360.4 | 166872.8 ± 19891.3 | 13202.2±1464.1 | 11787.5±1567.3 | 23811,9±4899,7 | 24935,8±279.7 |
| 13:0 | 689098.2 ± 43598.1 | 4914.8 ± 1307.2 | 63995.3 ± 22705.6 | 69681.2 ± 1449.5 | 6410.2± 493.7 | 11078.8±2364.1 | 10758.7±356.9 | 16592.8±380.1 |
| 14:1 *trans* | 871240.1 ± 147912.9 | 16799.5 ± 909.8 | 16341.6 ± 2119.1 | 38957.1 ± 38762.7 | 4226.5±1320.4 | 9403.6±5055.8 | 9348.1±1884.9 | 9885.5±618.8 |
| 14:1 *cis* | 587794.1 ± 38128.9 | 28773.7 ± 553.9 | 28198.5 ± 1369.7 | 62652.5 ± 58927.8 |  |  |  |  |
| 14:0 | 6563374.0 ± 338287.8 | 364867.5 ± 6158.6 | 295031.8 ± 35775.7 | 588279.3 ± 137829.0 | 107526.8±15036.6 | 119112.0±27970.4 | 129665.3±32171.4 | 122087.8±6898.7 |
| 15:1 | 1454605 ± 662249.8 | 18630.4 ± 596.0 | 27590.6 ± 3000.2 | 47915.3 ± 34530.5 | 14255.9±44.1 | 16399.9±2260.3 | 18591.9±5939.1 | 17998.9±273.1 |
| 15:0 | 2255903.0 ± 137724.3 | 86343.7 ± 31262.9 | 159972.7 ± 47559.6 | 545751.2 ± 5841.4 | 40310.3±371.3 | 59728.9±15503.0 | 68857.8±17544.7 | 72518.1±2272.6 |
| 16:1 | 4414113.0 ± 349803.2 | 1830497.0 ± 46063.1 | 1243368.1± 13055.9 | 4879306.0 ± 626427.8 | 49500.2±1409.7 | 73221.4±25473.6 | 68731.3±17493.5 | 169989.7±1039.9 |
| 16:0 | 98615312.0 ± 20960453.0 | 10417237.0 ± 917997.8 | 5525402.5 ± 671814.4 | 15038132.0 ± 1200688.0 | 3000952.3 ±286282.0 | 2280464.9±294792.1 | 3680092.2±890147.4 | 2792460.1±240794.1 |
| 17:1 | 1488676.0 ± 109214.2 | 5199.8 ± 879.7 | 139234.9 ± 15963.8 | 972905.4 ± 144027.0 | 25932.1±1620.6 | 54353.1±25898.3 | 36312.5±9426.2 | 71616.65386±3471.4 |
| 17:0 | 7293736.0 ± 1562205.0 | 222852.1 ± 96848.6 | 256008.6 ± 35400.0 | 2074079.0 ± 51235.2 | 85288.3±1779.9 | 70274.4±22047.6 | 128222.6±32013.3 | 117985.7±1085.1 |
| 18:3 | 6442834.0 ± 825966.6 | 19397.8 ± 7209.4 |  |  |  |  |  |  |
| 18:2 | 58707679.0 ± 4358033.9 | 647487.6 ± 9223.4 |  |  |  |  |  |  |
| 18:1 | 30208089.0 ± 1538399.2 | 1571038.0 ± 105418.4 | 9053235.9 ± 9830557.7 | 34325692.0 ± 4409892.4 | 165056.8±1502.3 | 153350.3±42745.4 | 308694.8±62800.2 | 316489.0±12740.0 |
| 18:0 | 1.63E+08 ± 50280110.6 | 8146139 ± 584602.6 | 3893004.1 ± 374594.2 | 19656507.0 ± 947558.1 | 2588244.3±177010.3343 | 2035457.6±160545.2 | 3076793.2±639128.0 | 2348955.3±67110.6 |
| 19:0 | 2303704.0 ± 162051.5 | 18869.9 ± 4791.8 |  |  |  |  |  |  |
| 20:2 | 241614.6 ± 53297.7 | 768.0 ± 177.4 |  |  |  |  |  |  |
| 20:1 | 2501343.0 ± 970901.5 | 39069.0 ± 5199.9 |  |  |  |  |  |  |
| 20:0 | 5443063.0 ± 83531.9 | 214798.8 ± 21012.7 |  |  |  |  |  |  |
| 22:1 | 648161.1 ± 37118.7 | 6606.2 ± 235.4 |  |  |  |  |  |  |
| 22:0 | 3335944.0 ± 118922.9 | 89891.7 ± 5906.2 |  |  |  |  |  |  |
| 24:1 | 342349.0 ± 140434.2 | 862.34 ± 131.3 |  |  |  |  |  |  |
| 24:0 | 8308666.0 ± 958699.7 | 71568.9 ± 11998.9 |  |  |  |  |  |  |
